# Synthesis of Substituted Oxo-Azepines by Regio- and Diastereoselective Hydroxylation

**DOI:** 10.3390/molecules22111871

**Published:** 2017-10-31

**Authors:** Harold Spedding, Peter Karuso, Fei Liu

**Affiliations:** Department of Molecular Sciences, Macquarie University, Sydney, NSW 2109, Australia; harold.spedding@hdr.mq.edu.au (H.S.); peter.karuso@mq.edu.au (P.K.)

**Keywords:** azepines, hydroboration, diastereoselectivity, oxo-azepines, DFT

## Abstract

Substituted seven-membered *N*-heterocycles are prevalent bioactive epitopes and useful synthons for preparing enzyme inhibitors or molecular recognition systems. To fully exploit the chemical properties of this flexible *N*-heterocycle scaffold, efficient methods for its diverse functionalization are required. Here we utilize the late-stage oxidation of tetrahydroazepines as an approach to access densely functionalized oxo-azepines in a total of 8 steps and ~30% overall yield from commercially available starting materials. Hydroboration of tetrahydroazepines proceeded with diastereoselectivity in a substrate-dependent manner to yield regioisomeric azepanols before their oxidation to the corresponding oxo-azepines. Regioselectivity of the hydroboration step may be improved moderately by a rhodium catalyst, albeit with loss of conversion to a competing hydrogenation pathway. Overall our method allows efficient access to azepanols and oxo-azepines as versatile epitopes and synthons with a high degree of diastereoselectivity and moderate regioselectivity.

## 1. Introduction

**Scheme 1:**
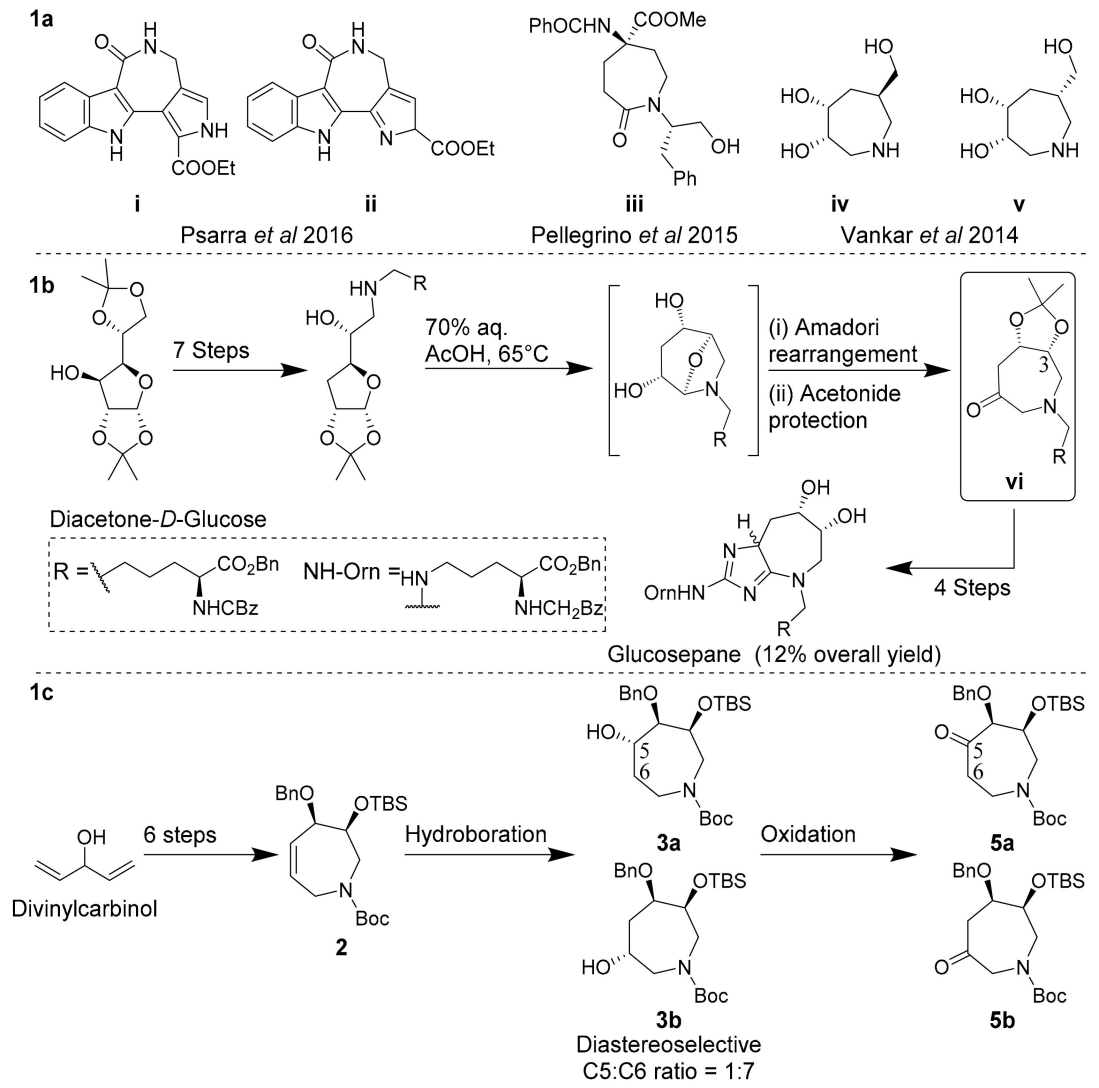
(**a**) Representative oxo-azepines as glycosidase inhibitors; (**b**) Concise, enantioselective synthesis of glucosepane by Spiegel et al. [24]; (**c**) Current work.

The seven-membered, *N*-heterocyclic azepane scaffold is a biologically active epitope and useful building block in the construction of novel glycosidase inhibitors [1,2,3,4,5], anticancers [6,7,8,9,10], antidiabetics [11,12,13,14,15], antivirals [16,17] and DNA minor groove-binding agents [18,19,20]. In particular, azepanols and oxo-azepines have received much recent attention as useful epitopes in both materials and medicinal chemistry due to the unique flexibility of the seven-membered ring and resultant spatial distribution of oxygen substituents. For example (Scheme 1a), 2-oxo-azepine **i** reported by Psarra et al. in 2016 exhibited a selective 78% inhibition of TAK1 kinase activity versus other kinase targets, while its constitutional isomer **ii** exhibited comparatively modest and non-selective inhibition of TAK1 activity (58%) [21]. Oxo-azepine **i** may now serve as a scaffold for a detailed structure-activity relationship (SAR) study towards optimized TAK1 inhibition. Pellegrino et al. enantioselectively synthesized and incorporated oxo-azepine **iii** into a tetrapeptide to induce a 3_10_ helical turn into the molecule, as confirmed by NMR and molecular dynamics [22]. This 3_10_ helical conformation was found to be stabilized by key intramolecular hydrogen bonds between the 2-oxo-azepine ketone oxygen and amino acid residues [22,23]. Vankar et al. in 2014 reported *syn*-dihydroxyazepane **iv** as a potent and selective inhibitor of jack bean α-mannosidase, while its epimer **v** was two orders of magnitude less active towards this enzyme [2]. In a recent landmark example, Spiegel et al. prepared glucosepane enantioselectively from oxo-azepine **vi**, yielding a guanidine-containing oxo-azepine (Scheme 1b) [24]. Collectively, these examples highlight the importance of stereochemically well-defined and highly functionalized azepanols and oxo-azepines as biologically active epitopes and synthons. These examples also highlight the need for efficient synthetic methods to access these molecules with regio- and diastereocontrol.

Azepanols and oxo-azepines are typically constructed via the ring-expansion or cyclization of appropriately derivatized sugars or furans with pre-established substitution patterns and stereochemistry. Methods of ring-expansion and cyclization have most-commonly included reductive amination [19,20,25,26,27,28,29] or epoxide ring opening with suitable amines [30,31], ring-closing metathesis (RCM) [32,33,34], alkene–nitrone cycloaddition [35] and tandem Staudinger aza–Wittig reactions [36]. The Spiegel synthesis of glucosepane utilized commercially available diacetone-d-glucose ($3.20/g) as a precursor to oxo-azepine **vi** via a key Amadori rearrangement (Scheme 1b) [24]. Oxo-azepine **vi** was then further elaborated via the C3-ketone with a sigmatropic rearrangement and cyclization sequence to yield glucosepane in a total of 13 steps and 12% overall yield from diacetone-d-glucose [24]. As part of our work on densely functionalized and conformationally diverse azepanes, we have sought to develop a more-concise route to access oxo-azepines via a late-stage oxidation of tetrahydroazepines [37,38]. Here we report the diastereoselective synthesis of useful oxo-azepine synthons that can be incorporated into complex oxo-azepines such as the glucosepanes (Scheme 1c).

Our previous work showed that hydroboration of the (3*S*)-azido-(4*R*)-tetrahydroazepine proceeded in low regio- and diastereoselectivity [37,38,39]. Herein, we investigate regio- and diastereoselectivity in the hydroboration of a related tetrahydroazepine **2** (Scheme 1c). Standard and catalytic hydroboration reactions yielded a mixture of the expected (5*S*) and (6*R*) alcohols **3a** and **3b**, respectively, with complete diastereofacial selectivity and moderate regioselectivity. These azepanols were then converted to the corresponding oxo-azepines **5a** and **5b** by oxidation. Overall, the current work with tetrahydroazepine **2** constitutes an efficient and shorter route (8 steps from commercially available simple starting material) to azepanols and oxo-azepines that can be used as synthons for complex oxo-azepines such as the glucosepanes (13 steps, 12% overall yield).

## 2. Results

### 2.1. Synthesis of Silyl Ether ***2***

**Scheme 2:**
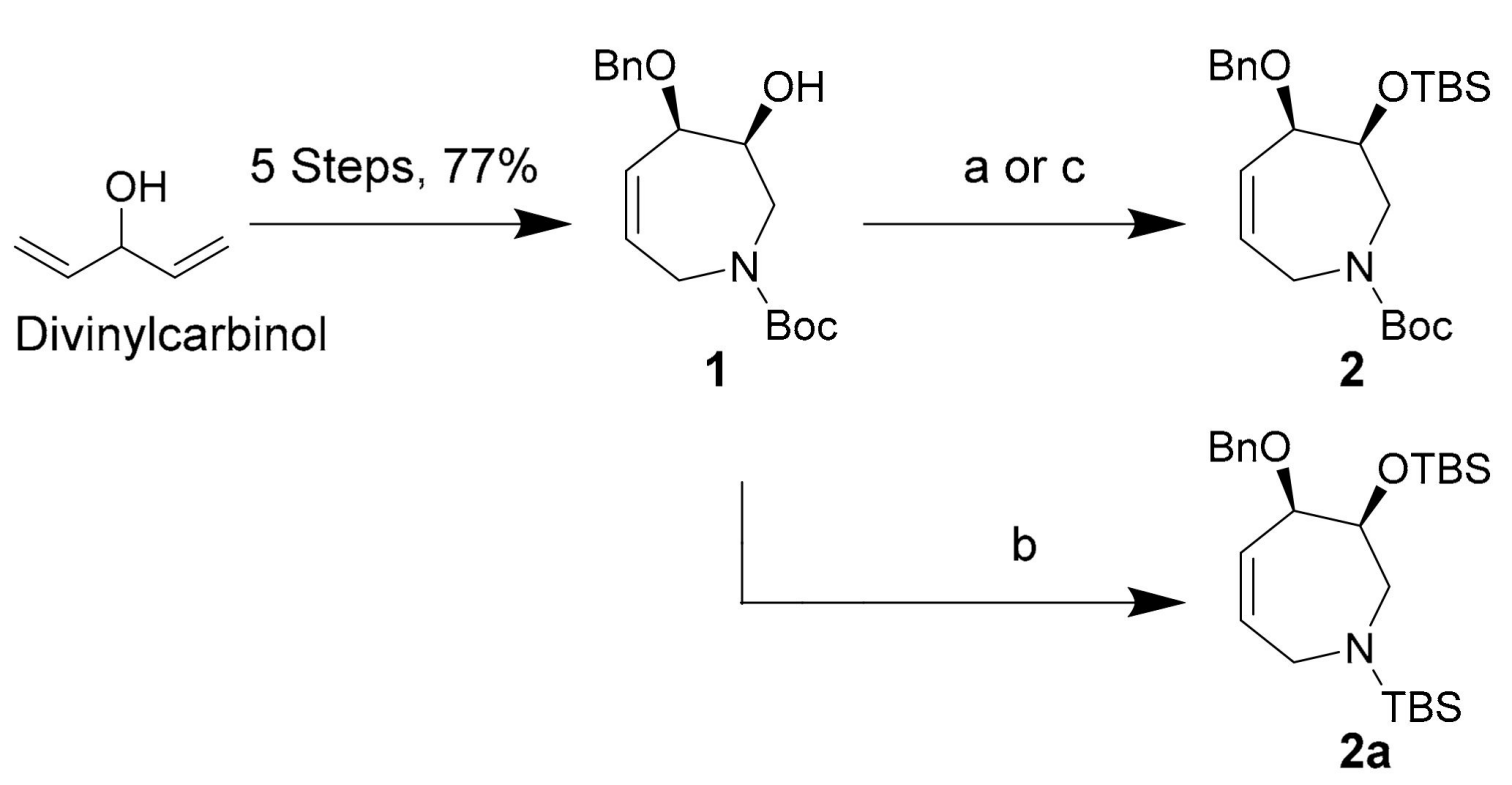
Silyl-protection conditions for secondary alcohol **1**. Conditions (a) TBSOTf (1.0 equiv), 2,6-lutidine (1.2 equiv), −80 °C, DCM, 2 h, 90%; (b) TBSOTf (1.0 equiv), 2,6-lutidine (1.2 equiv), −20 °C, DCM, 2 h, 50% (of **2a**); (c) TBSCl (3.0 equiv), imidazole (3.5 equiv), 35 °C, DMF, 24 h, 30%.

Tetrahydroazepine starting material **1** was synthesized from commercially available divinylcarbinol using a concise, 5-step protocol described by Fürstner et al. in 77% overall yield [32]. Silyl ether **2** was then produced in 90% yield using Corey’s TBSOTf conditions over 2 h at −80 °C (Scheme 2, conditions (a)) [40]. The same reaction conducted at −20 °C led to *N*-Boc deprotection of **1** and to the formation of *O,N*-TBS protected species **2a** according to preliminary NMR spectroscopic data (Scheme 2, conditions (b)). Compound **2a** appears to degrade by *N*-TBS hydrolysis over several days at −20 °C. The use of Corey’s conditions with TBSCl and imidazole in DMF resulted in long reaction times and poor yields for our sterically congested substrate **1** (Scheme 2, conditions (c)), with only 30% conversion to **2** after 48 h at 35 °C [41].

### 2.2. Hydroboration of (3S)-OTBS Tetrahydroazepine ***2***

**Scheme 3:**
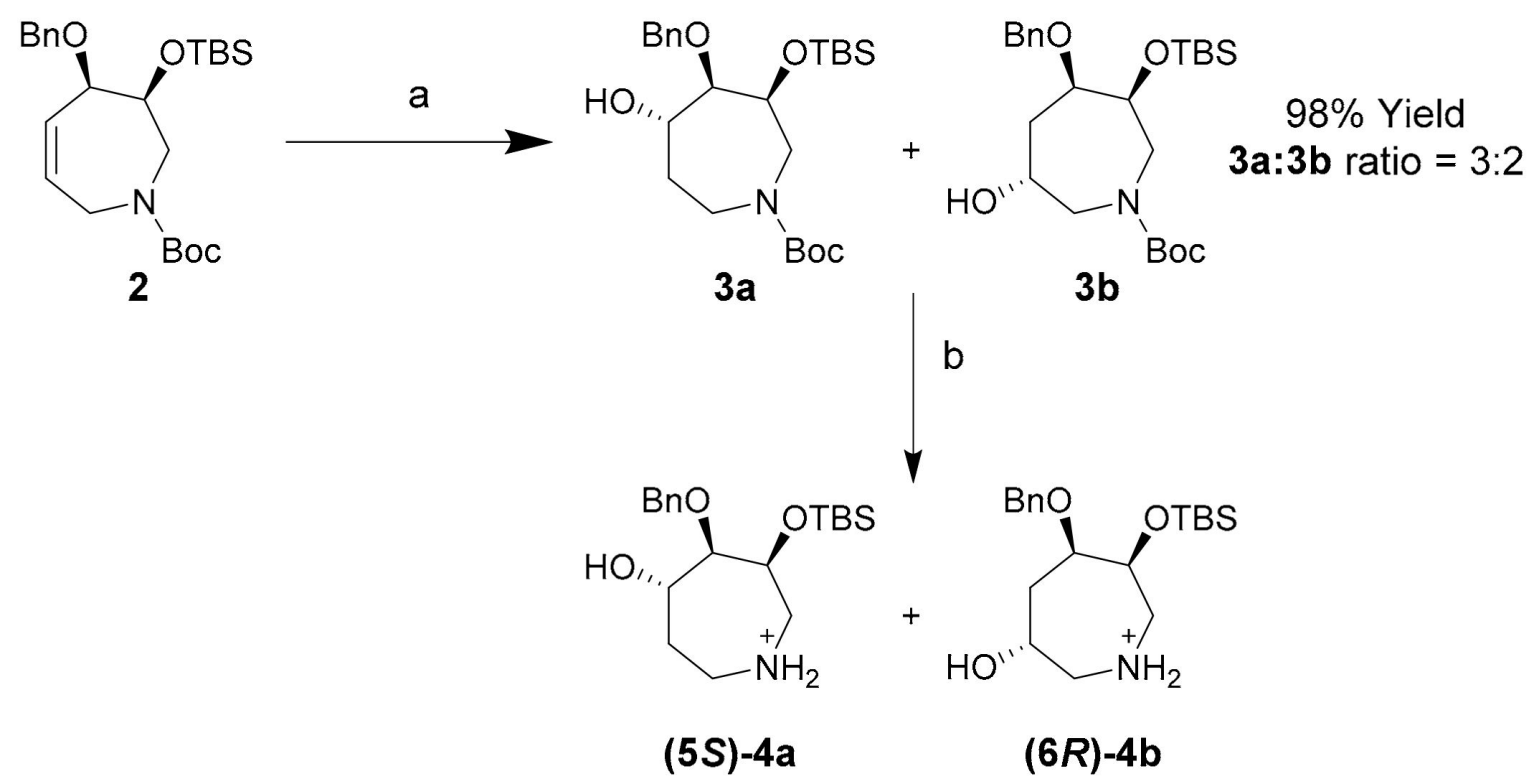
Synthesis of azepanols **3a** and **3b** and their deprotected derivatives **(5*S*)-4a** and **(6*R*)-4b**. Conditions (a) (i) BH_3_∙SMe_2_, THF, r.t., 1 h; (ii) NaOH, H_2_O_2_, EtOH, 80 °C, 1 h, 92%; (b) TFA, r.t., 5 min, 99%.

Hydroboration of tetrahydroazepine **2** proceeded smoothly to yield a 3:2 mixture of regioisomers **3a** and **3b** with excellent diastereofacial selectivity and high yield utilizing borane dimethylsulfide (BH_3_∙SMe_2_) (Scheme 3). These compounds were isolated by semi-preparative reverse-phase HPLC using acetonitrile:water:trifluoroacetic acid (TFA) (from 50:50:0.1 to 80:20:0.1 over 45 min; flow rate: 4.18 mL/min) as the mobile phase. Separated regioisomers **3a** and **3b** were de-protected to yield azepanols **(5*S*)-4a** and **(6*R*)-4b** for characterization by 2D-NMR in the absence of *N*-Boc rotamers. 

### 2.3. Structural Assignment of ***(5*S*)-4a*** and ***(6*R*)-4b***

**Figure 1:**
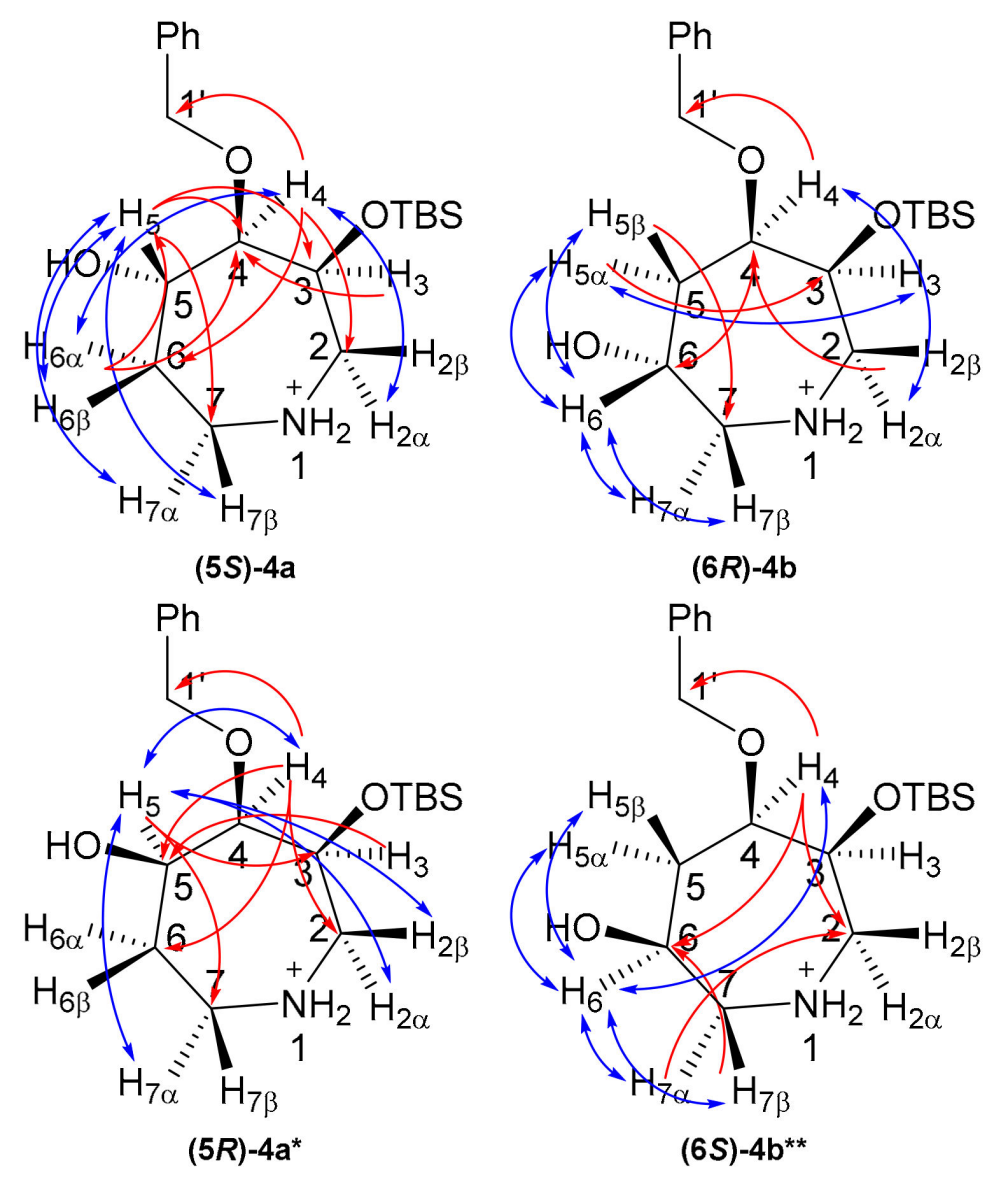
2D-NMR correlations for azepanols **(5*S*)-4a** and **(6*R*)-4b**. Red, HMBC; blue, NOESY. * Characterized as a mixture with **(5*S*)-4a**; ** Characterized as a mixture with **(6*R*)-4b**.

Key 2D-NMR correlations (Figure 1) were used to completely assign the ^1^H and ^13^C resonances (see Supplementary Materials). Briefly for **(5*S*)-4a**, an HMBC correlation between C1′ (δ_C_ 74.0 ppm) and H4 (δ_H_ 3.55 ppm), in combination with an HSQC correlation between H4 and C4 (δ_C_ 85.6 ppm) was used to identify C4/H4. COSY correlations and HSQCs were then used to assign the rest of the azepine ring (Table S1). NOESY correlations between H4/H6α and H4/H2α allowed stereochemical assignment of H6 and H2 protons. The presence of a strong nOe correlation between H5 and H6β and the absence of nOe correlations between H5 and H3 or H2α suggested 5*S* stereochemistry at the site of hydroxylation. To further substantiate this hypothesis, compound **(5*S*)-4a** was synthesized by oxidation of **(5*S*)-4a** to the 5-ketone **5a**, followed by reduction to a mixture of **(5*S*)-4a** and **(5*S*)-4a**. A **(5*S*)-4a**:**(5*R*)-4a** ratio of 4:1 was observed for this reduction, likely due to the steric bulk of the *re* face of the azepine ring leading to the observed diastereofacial selectivity in the hydroboration reaction. For **(5*R*)-4a**, nOe correlations from H5 to H4 and H7α and an absence of nOe correlations between H5 and H7β (Table S2) suggested 5*R* stereochemistry for the minor component, but the NMR data was far from convincing due to overlapping peaks, especially for the C2 and C7 protons. DFT calculations (next section) were used to substantiate the stereochemical assignments.

Compound **(6*R*)-4b** was characterized by the same approach. The combination of an HMBC between C1′ (δ_C_ 72.5 ppm) and H4 (δ_H_ 3.84 ppm), and an HSQC between H4 and C4 (δ_C_ 77.5 ppm) was used to identify C4/H4. COSY correlations and HSQCs were used to identify the remainder of the azepine ring and site of hydroxylation (C6; 63.7 ppm, Table S3). The 6*R* stereochemistry of **(6*R*)-4b** was supported by strong nOe correlations from H6 to H5α, H5β, H7α and H7β. This suggested that H6 is synclinal to H5α, H5β, H7α and H7β, but this could not be used to confirm the stereochemistry. Similarly, the absence of nOe correlations from H6 to H2α, H3 and H4 was weak evidence of **6*R*** stereochemistry. To help with the assignment, a mixture of **(6*R*)-4b** and **(6*S*)-4b** was synthesized using the same oxidation/reduction approach as for **4a** (Table S4). In compound **(6*S*)-4b**, H6 showed stronger nOe correlations to H5α and H7α than to H5β and H7β, consistent with a β-OH and thus 6*S* stereochemistry for this compound and in support of 6*R* stereochemistry for **4b**.

### 2.4. DFT Analysis of ***(5*S*)-4a*** and ***(5*R*)-4a***

**Figure 2:**
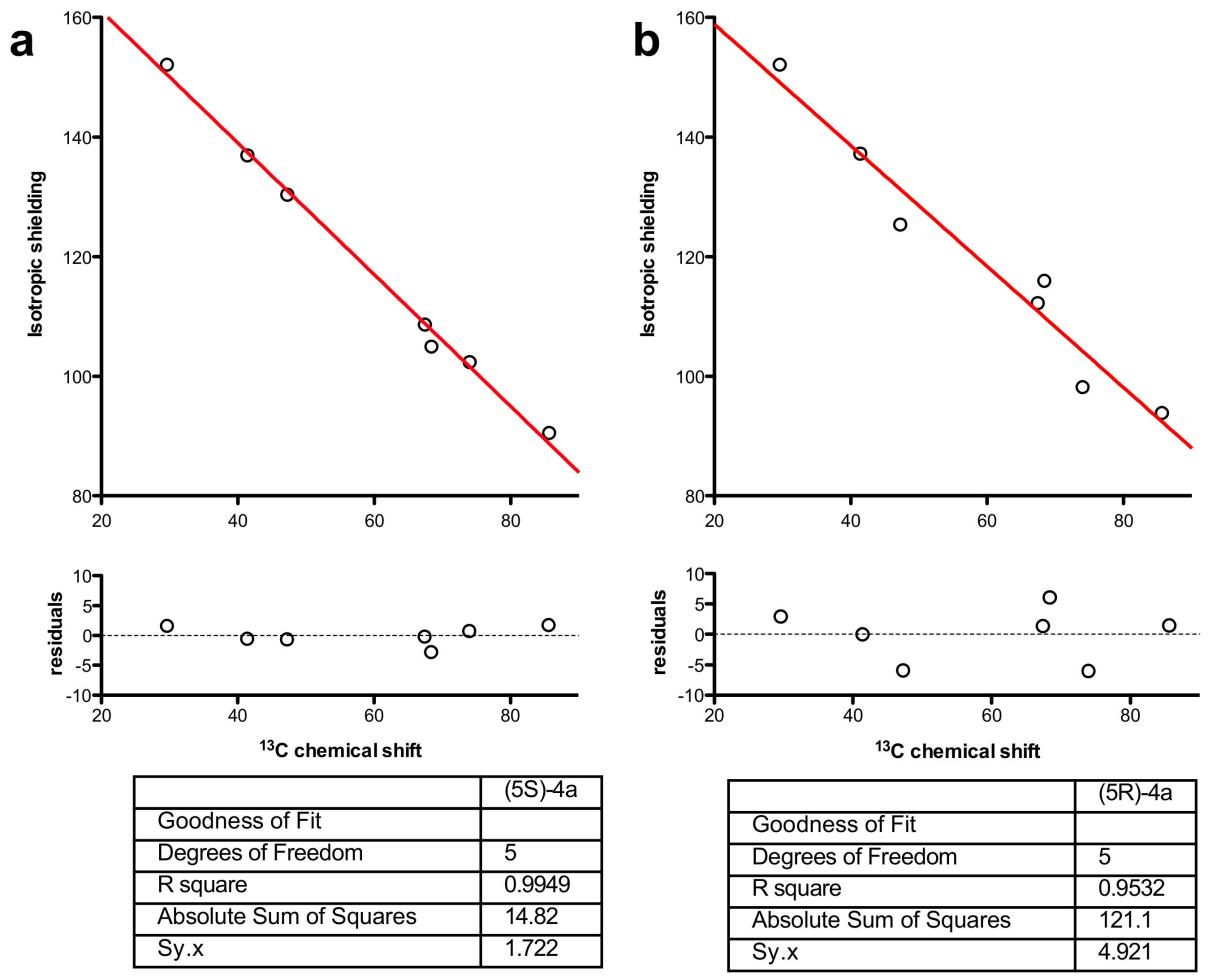
Non-linear regression fitted to a straight line of chemical shifts against calculated isotropic shielding (DFT//B3-LYP/TZVPP) for (**a**) **(5*S*)-4a** and (**b**) **(5*R*)-4a** with residuals and goodness-of-fit values.

Models of **(*5S*)-4a** and **(5*R*)-4a** were built in MOE (Molecular Operating Environment) [42], and a conformational search using low-mode molecular dynamics was made from each structure and the remaining azepine geometry using the MMFF94x force-field for minimization. The TBS and Ph groups were then stripped from the structures and the remaining azepines’ geometry optimized using Turbomole 7.1 (DFT//BP86/SV(P) then DFT//B3-LYP/def2-TZVPP) [43]. The NMR shielding constants were then calculated at the same level of theory (DFT//B3-LYP/def2-TZVPP) and plotted against the actual ^13^C- and ^1^H-NMR data of **(5*S*)-4a** and **(5*R*)-4a** (see Supplementary Materials). Non-linear least-squares fitting to a straight line (GraphPad Prism 5.0) resulted in a goodness of fit (R; Figure 2).

**Figure 3:**
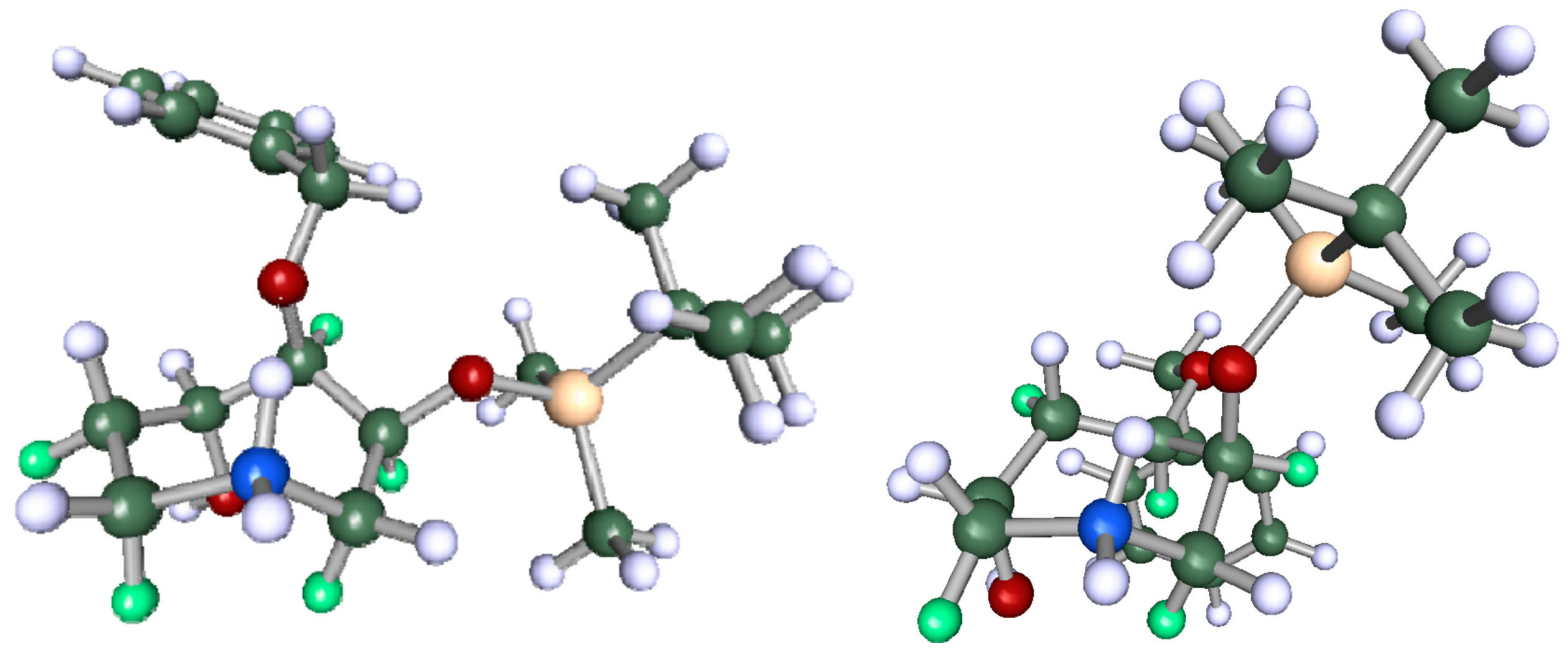
Global lowest-energy structure of **(5*S*)-4a** (**left**) and **(6*R*)-4b** (**right**), α-protons coloured green.

Non-linear regression showed a better fit for ^13^C-NMR chemical shifts for **(5*S*)-4a** (Figure 2a) than for **(5*R*)-4a**, whereas the lowest-energy structure gave a poorer fit (R^2^ = 0.9532 vs. 0.9949) with several ^13^C resonances (notably C5 and the benzylic carbon) greater than 5 ppm from the line of best fit (Figure 2b). In light of this regression analysis and experimental spectral data for **4a**, we conclude that the molecule has, on the weight of evidence, 5*S* stereochemistry. Finally, it is noteworthy that the DFT calculations predict a conformation (Figure 3) where H5 would not show any diagnostic nOes to H3 and H2α. Similarly, the minimum-energy structure for **(6*R*)-4b** (Figure 3, right) showed that H5 is synclinal to H5α, H5β, H7α and H7β, and that H5 would indeed not be expected to show nOe correlations to H2α, H3 or H4 and nOes of roughly equal intensity to H5α, H5β, H7α and H7β, as is observed (see Supplementary Materials).

### 2.5. Reagent and Catalyst Screen for the Hydroboration of ***2***

**Scheme 4:**
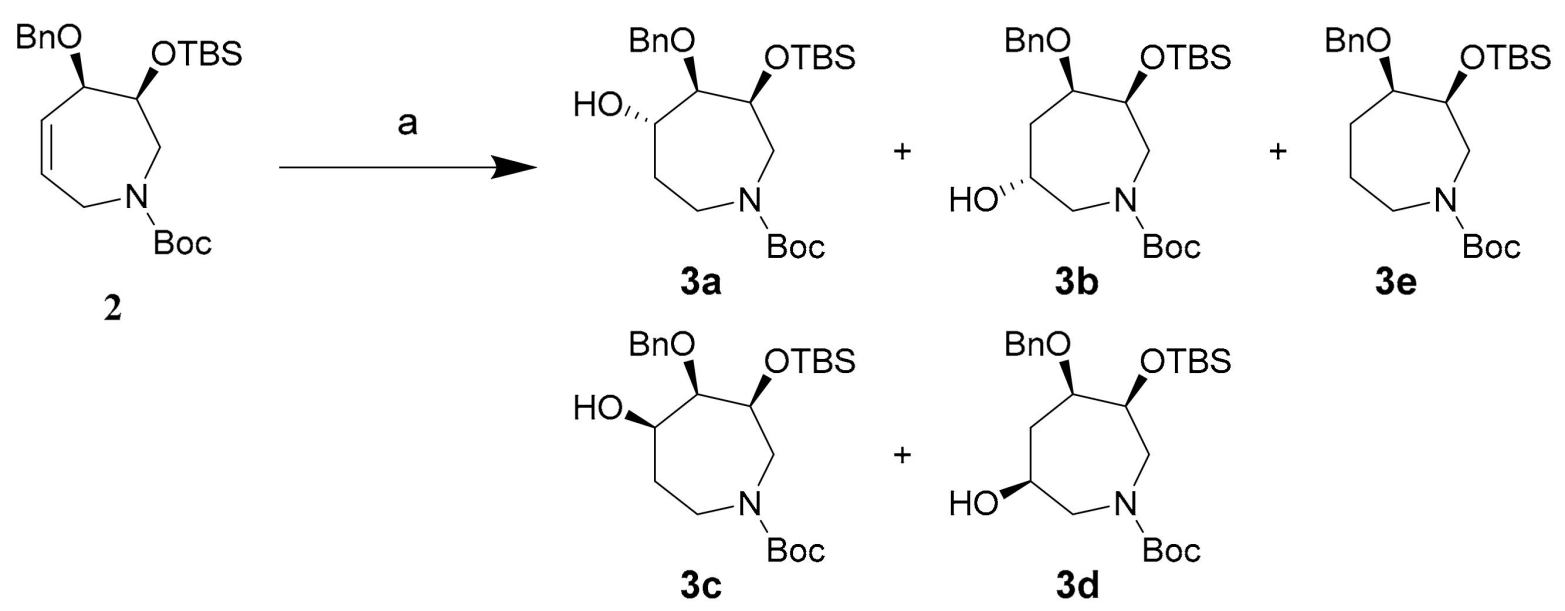
Reagent and catalyst screen for the hydroboration of **2**. Conditions (a) (i) BHR_2_, see Table 2, THF; (ii) NaOH, H_2_O_2_, EtOH, 80 °C, 1 h, 99%.

The stereochemical and regiochemical outcomes of hydroborations of substituted alkenes is related to the steric and stereoelectronic environment of both the reagent and substrate. In this case, the hydroboration of (3*R*)-OTBS tetrahydroazepine **2** using BH_3_∙SMe_2_ proceeded with excellent diastereofacial selectivity but low regioselectivity to provide **3a** and **3b**. In contrast, hydroboration of a (3*S*)-azidotetrahydroazepine in our earlier work proceeded with low diastereo- and regioselectivity, yielding all four possible C5/C6 azepanols (product ratio 5*S*:5*R*:6*S*:6*R* = 1:36:32:28) [39]. It is likely that the bulky (3*R*)-OTBS group, along with the other substituents of tetrahydroazepine **2**, present significant steric bias to the cyclic alkene that was absent in the (3*S*)-azido substrate. We were thus interested in screening reagents and catalysts to explore potential regioselectivity in the hydroboration of substrate **2** (Scheme 4).

Hydroboration of **2** with a variety of common borylating reagents (Table 1) proceeded with poor or no yield of the azepanols (Table 1, Entries 2–7) except for BH_3_∙SMe_2_ (Table 1, Entry 1). The use of more-stable borylating reagents, including catechol borane, pinacol borane and 9-BBN, yielded no azepanol product upon oxidation, even when borylation was conducted in refluxing THF. In the case of catechol borane, the addition of Wilkinson’s catalyst (Table 1, Entry 5) resulted exclusively in the formation of hydrogenation product **3e**.

Transition-metal catalysts for hydroborations have been shown to exhibit remarkable chemo- [44], regio- [45] and stereoselectivities [44,46,47,48,49] for a variety of acyclic and cyclic substrates [44,50,51,52,53]. We screened a variety of catalysts for the hydroboration of tetrahydroazepine **2** (Table 2). Use of iridium and rhodium catalysts led to the formation of hydrogenation product **3e** as the major product (Table 2, Entries 1–3). This suggests the addition of our densely functionalized substrate, **2**, to the metal centre is slow, as expected for unreactive olefins [54,55]. Loading of **2** is likely out-competed by diboration of the metal centre with accompanying hydrogen release that leads to formation of **3e** in the presence of the metal catalyst [54,55].

Due to the difficulty in isolating hydrogenation product **3e** from starting material **2** by semi-preparative LC, **3e** was formed directly by hydrogenation of compound **2**. The ^1^H-NMR spectrum of compound **3e** was then compared with crude reaction mixtures (Table 2, Entries 1–2) to verify its formation under catalytic hydroboration conditions (for chromatograms, see Supplementary Materials). Compound **3e** was deprotected to yield **4c** and characterized by 2D-NMR in the absence of *N*-Boc rotamers (Table S5).

In the case of PinBH with Rh(COD)(DPPB)BF_4_, modest conversion to the desired azepanol products was observed with a considerable bias towards the 6*R* regioisomer **3b** while preserving the diastereoselectivity (Table 2, Entry 2). Improved regioselectivity may be accounted for by borylation at the most sterically accessible position, as is observed with other OTBS-protected substrates. This steric effect appears to outweigh any potential directed borylation via the allylic OBn group to the *syn* face of the substrate. Addition of the Lewis acid additive tris(pentafluorophenyl)borane (FAB), as reported by Lata and Crudden [56], increased the formation of **3e** without improving the regioselectivity between **3a** and **3b** (Table 2, Entry 3). The use of a copper (I) metal centre with TangPhos (Table 2, Entry 4) and bulky (*R*)-DTBM SegPhos (Table 2, Entry 5) ligands resulted in no formation of any product [57,58].

In contrast to neutral rhodium catalysts for hydroboration, rhodium (I) catalysts exhibit marked solvent-dependent variations in yield, stereo- and regioselectivity for a variety of substrates [52,54,55]. In an attempt to optimize the hydroboration of **2** with PinBH/5 mol % Rh(COD)(DPPB)BF_4_, the most-active catalyst for the substrate, a solvent screen was conducted (Table 3). In the relatively high-polarity solvent DCE at 60 °C, hydroboration proceeded with good conversion and suppression of the hydrogenation product **3e** (Table 3, Entry 1) compared to the same reaction at ambient temperature (Table 2, Entry 2). This reaction also proceeded with comparable diastereoselectivity and an increase in regioselectivity (Table 3, Entry 1, **3a**:**3b** = 1:8) in favour of **3b** as the kinetic product compared to the same reaction at ambient temperature (Table 2, Entry 2, **3a**:**3b** = 1:7).

In THF and toluene, the reaction proceeded with the highest regioselectivity (**3a**:**3b** = 1:8, Table 3, Entry 5) in favour of **3b** with similarly high conversion upon heating (Table 3, Entries 4 and 6). In these solvents, THF exhibited the least formation of hydrogenation product **3e**, while toluene exhibited the most. Considering that DCE and toluene, the most- and least-polar of the solvents screened, respectively, had comparable amounts of hydrogenation product **3e**, the formation of **3e** does not appear to be dependent on solvent polarity. It may be possible, then, that the ability of THF to coordinate the borane and metal centre precludes diboration of the metal centre and therefore mildly suppresses formation of **3e**. In addition, reduction of the catalyst loading from 5–10 mol % to 1 mol % in DCE (Table 3, Entry 2) increased the proportion of **3e** formed, suggesting borane addition to the metal centre effectively out-competes alkene addition. In toluene (Table 3, Entry 7), reduction of the catalyst loading in the same manner yielded an 18% decrease in the amount of **3e** formed, although conversion was still poor over 18 h. In summary, hydrogenation is competitive with hydroboration of our very sterically congested azepane substrate **2**. As a compromise between conversion, regioselectivity and suppression of **3e** formation, the optimal conditions for the catalytic hydroboration of **2** with PinBH/5 mol % Rh(COD)(DPPB)BF_4_ is THF at 60 °C (Table 3, Entry 4). These conditions facilitate nearly complete conversion of substrate **2** to azepanol regioisomers **3a** and **3b** in a ratio of 1:7, with minimal formation of hydrogenation product **3e**.

### 2.6. Synthesis and Structural Assignment of (3R)-OTBS-5/6-Oxo-Azepines ***5a*** and ***5b***

**Scheme 5:**
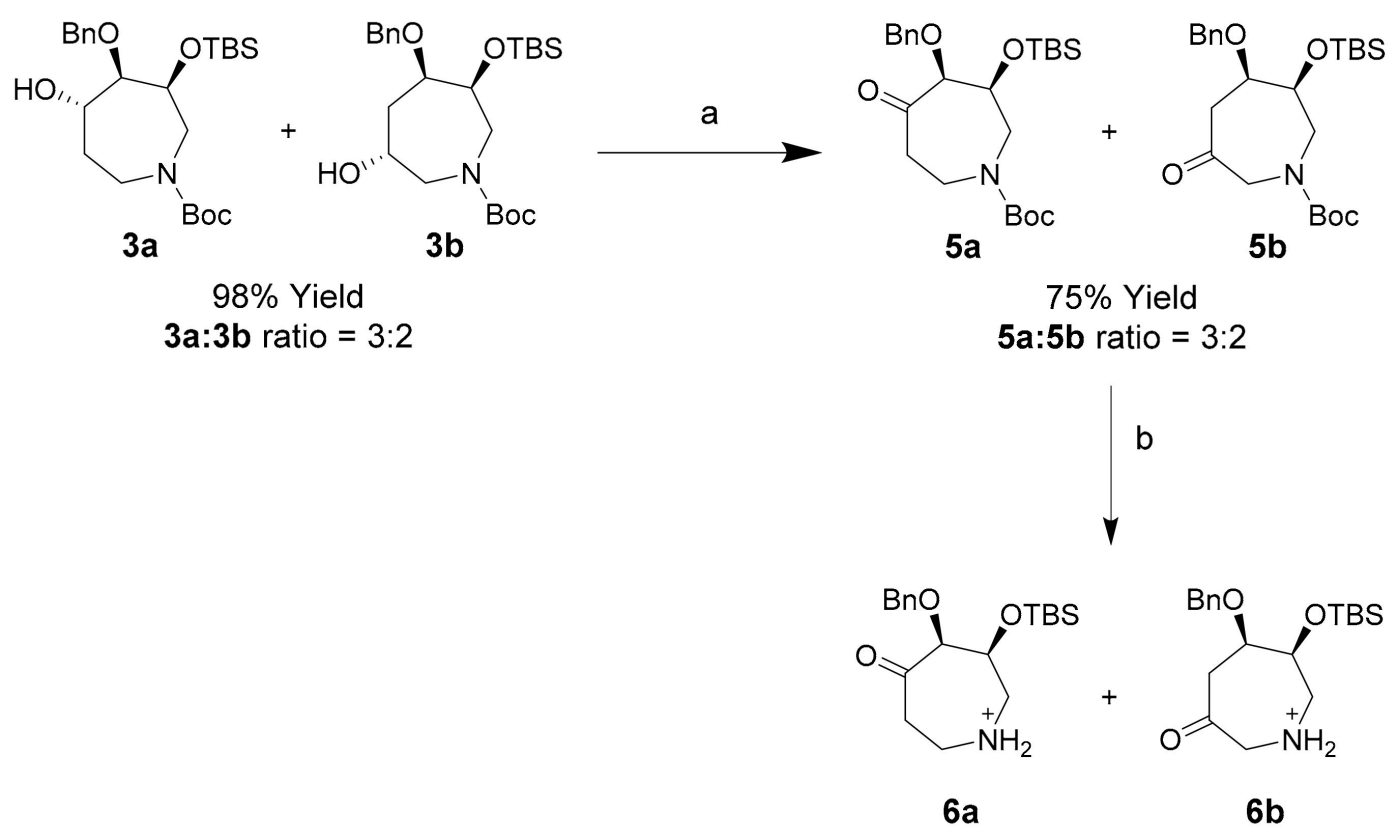
Synthesis of oxo-azepines **5a** and **5b** and their *N*-Boc deprotected counterparts **6a** and **6b**. Conditions (a) PCC, DCM, 25 °C, 45 min, 75%; (b) TFA, r.t., 5 min, 99%.

Oxidation of crude **3a**/**3b** azepanol mix using pyridinium chlorochromate proceeded to completion in 1 h, however in modest yield of oxo-azepines **5a** and **5b** (Scheme 5). Hydration of these products during work-up resulted in loss during column chromatography on Celite and silica gel. This problem was circumvented using dried 4Å molecular sieves (MS) for work-up, improving the combined isolated yield of **5a** and **5b** to 75%. *N*-Boc deprotected derivatives **6a** and **6b** were characterized by 2D-NMR spectroscopy.

**Figure 4:**
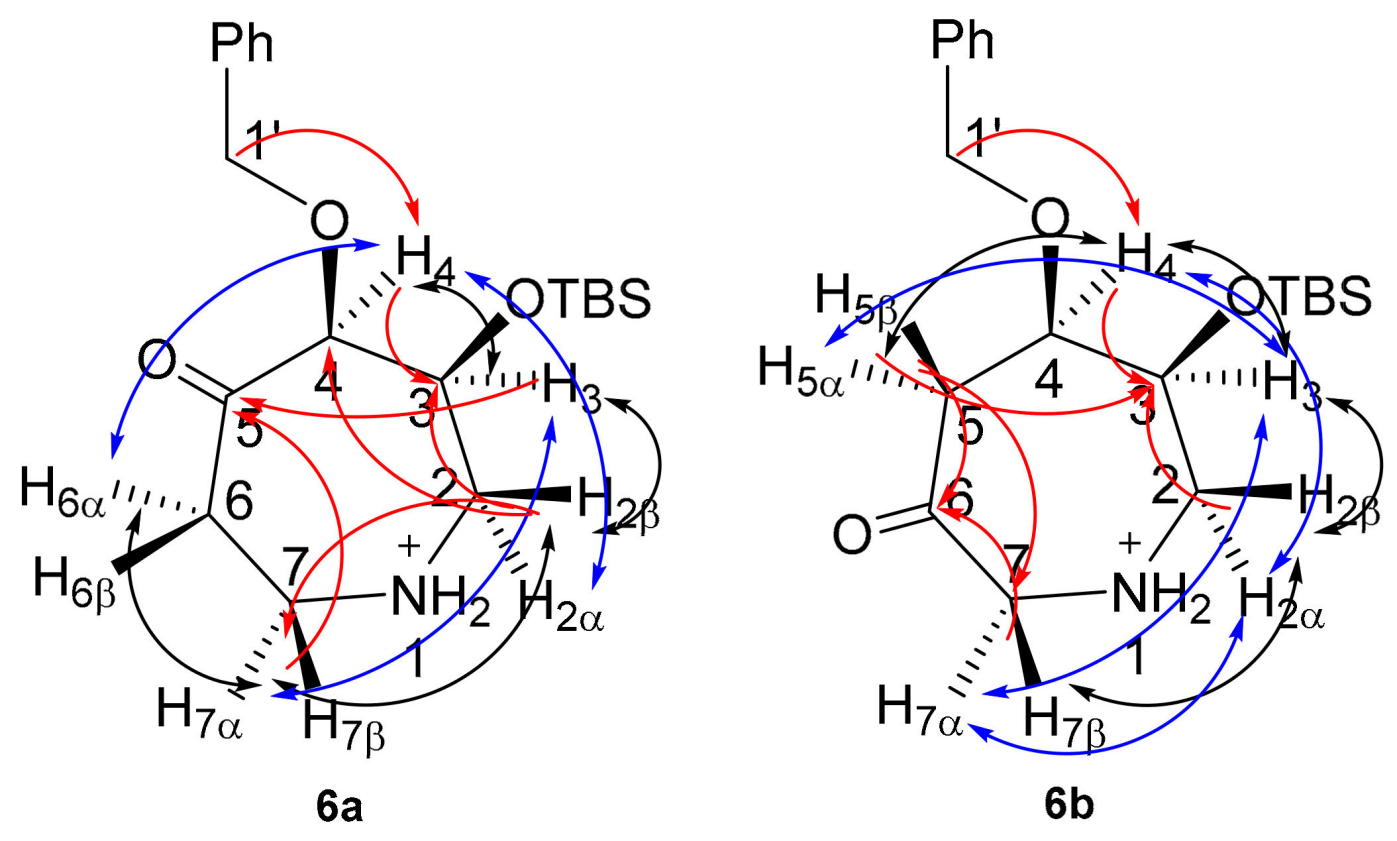
2D-NMR correlations for monohydroxyl azepanes **6a** and **6b**. Red, HMBC; black, COSY; blue, NOESY.

In comparison to azepanols **(5*S*)-4a** and **(6*R*)-4b**, structural assignment of ketones **6a** (Table S6) and **6b** (Table S7) is considerably less ambiguous in the absence of the monoalcohol stereocentre. These ketones, with their characteristic carbonyl ^13^C chemical shift, can be assigned directly from HMBC correlations and indirectly by a combination of COSY and HSQC correlations. In addition, the stereoelectronic bias exerted by the ketone reduces the conformational freedom of the ring, enhancing observed COSY and NOESY correlations (Figure 4).

For **6a**, an HMBC between H1’ (δ_H_ 4.47, 4.85 ppm) and C4 (δ_C_ 87.2 ppm), combined with an HSQC between C4 and H4 (δ_H_ 4.32 ppm), was used to identify C4 (Table S6). In the same manner, COSY and HSQC correlations were used to assign the rest of the azepine ring (Table S6). Strong HMBC correlations from C5 (δ_C_ 202.1 ppm) to H3, H7α and H7β were used to identify the ketone at C5. 

For **6b**, an HMBC between H1’ (δ_H_ 4.67 ppm) and C4 (δ_C_ 75.5 ppm), combined with an HSQC between C4 and H4 (δ_H_ 3.84 ppm), was used to identify C4. COSY correlations and HSQC were then used to assign the rest of the azepine ring (Table S7). A strong ^3^*J*_CH_ correlation from H4 to C6 (δ_C_ 200.5 ppm) and ^2^*J*_CH_ correlations from H5α, H5β, H7α and H7β to C6 were used to identify the ketone at C6. Stereospecific proton assignments were based on nOe correlations (Tables S6 and S7).

## 3. Materials and Methods

### 3.1. General Methods

All reagents were purchased from Sigma-Aldrich and used without further purification. Dichloromethane (DCM) and tetrahydrofuran (THF) were distilled from calcium hydride. All reactions were conducted in flame-dried glassware and under nitrogen atmosphere unless stated otherwise. All reactions were magnetically stirred and monitored by thin-layer chromatography (TLC) using Merck silica gel 60 F_254_ pre-coated plates (0.25 mm). Flash column chromatography was performed using silica gel (0.032–0.063 mm particle size) from Fisher Scientific. All ^1^H-, ^13^C- and 2D-NMR experiments were performed on either a Bruker Avance DPX 400 MHz, Bruker Avance III HD NanoBay 400 MHz or DRX600 NMR spectrometer equipped with a TXI (5 mm) cryoprobe. Chemical shifts were reported in ppm using residual CDCl_3_ (δ_H_; 7.26 ppm, δ_C_; 77.36 ppm), or (CD_3_)_2_CO (δ_H_; 2.05 ppm, δ_C_; 29.84 ppm), or CD_3_OD (δ_H_; 3.31 ppm, δ_C_; 49.00 ppm) as an internal reference. Polarimetry data was collected on a JASCO polarimeter (model no. P-1010). LC–MS was conducted using an Agilent 6130 single-quadrupole LC–MS system. High-resolution mass-spectrometry (HRMS) was conducted using a Thermo-Fisher QExactive-Plus Orbitrap instrument. Infrared spectroscopy was conducted using a Thermo-Fisher Nicolet iS5 FT–IR spectrometer.

### 3.2. Synthetic Procedures

#### 3.2.1. *tert*-Butyl(3*S*,4*R*)-4-(benzyloxy)-3-((*tert*-butyldimethylsilyl)oxy)-2,3,4,7-tetrahydro-1*H*-azepine-1-carboxylate (**2**)

To a stirred solution of tetrahydroazepanol **1** (0.571 g, 1.8 mmol) in dry DCM (8.9 mL) at −40 °C was added successively 2,6-lutidine (0.497 mL, 4.3 mmol) and *tert*-butyldimethylsilyl trifluoromethanesulfonate (0.822 mL, 2.1 mmol). The reaction proceeded at the same temperature for 40 min until completion according to TLC before being quenched by the addition of H_2_O. The organic layer was extracted twice with DCM, dried with MgSO_4_ and concentrated in vacuo. Crude residue was purified by flash chromatography (petroleum ether/ethyl acetate 9:1) to yield **2** as a clear, light yellow oil (90%). ${\left[\alpha \right]}_{D}^{20}$ –43 (*c* 0.19, CHCl_3_); FTIR (ATR): 2953, 2929, 1697, 1411, 1245, 1169, 1137, 834, 776 cm^−1^. ^1^H-NMR (400 MHz, CDCl_3_): δ 7.37–7.23 (m, 5H), 5.93–5.62 (m, 2H), 4.67–4.54 (m, 2H), 4.28–4.24 (m, 1H), 4.15–4.06 (m, 1H), 3.99–3.81 (m, 2H), 3.73–3.62 (m, 1H), 3.33–3.23 (m, 1H), 1.41 (s, 9H), 0.89 (s, 5H), 0.87 (s, 4H), 0.07–0.06 (m, 6H). ^13^C-NMR (150 MHz, CDCl_3_): δ 138.94, 130.93, 129.43, 128.61, 127.88, 127.76, 78.22, 72.26, 71.60, 51.46, 46.29, 28.76, 26.17, 18.52, −4.2418, −4.50. HRMS–ESI: [M + H]^+^ calcd. for C_24_H_40_NO_4_Si: 434.2727; found 434.2718.

#### 3.2.2. *tert*-Butyl(3*S*,4*R*,5*S*)-4-(benzyloxy)-3-((*tert*-butyldimethylsilyl)oxy)-5-hydroxyazepane-1-carboxylate (**3a**); *tert*-Butyl(3*S*,4*R*,6*S*)-4-(benzyloxy)-3-((*tert*-butyldimethylsilyl)oxy)-5-hydroxyazepane-1-carboxylate (**3b**)

To a solution of silyl ether **2** (142 mg, 0.33 mmol) in THF (316 µL) at 25 °C and under N_2_ atmosphere was added BH_3_∙SMe_2_ (2.0 M in THF, 180 µL, 0.36 mmol) dropwise. The reaction mixture was left to stir at the same temperature for 1 h before sequential addition of ethanol (1.92 mL), 6 M sodium hydroxide (1.44 mL) and 30% H_2_O_2_ (0.67 mL), followed by heating to 60 °C for 1 h. The reaction was cooled and quenched by the addition of saturated NH_4_Cl, after which the layers separated. The aqueous layer was then extracted with ethyl acetate (3 × 5 mL), dried with MgSO_4_ and rotary evaporated to yield crude alcohols **3a** and **3b** (136 mg, 92% yield) as a pale yellow oil. Crude product was used for PCC oxidation without purification. In addition, preparative HPLC (mobile phase—acetonitrile:water:TFA from 50:50:0.1 to 80:20:0.1 over 45 min; flow rate–4.18 mL/min) was used to isolate regioisomeric alcohols **3a** and **3b** as colourless oils with an overall yield of 92% and **3a**:**3b** ratio of 3:2. 

(**3a**) ${\left[\alpha \right]}_{D}^{20}$ +19 (*c* 0.02, CHCl_3_). FTIR (ATR): 2953, 2929, 1671, 1200, 1132, 835, 721 cm^−1^. ^1^H-NMR (400 MHz, CDCl_3_): δ 7.40–7.26 (m, 5H), 4.78–4.70 (m, 1H), 4.55–4.40 (m, 1H), 4.40–4.26 (m, 1H), 4.06–3.96 (m, 1H), 3.80–3.40 (m, 3H), 3.34–3.15 (m, 2H), 2.58 (s, broad, 1H), 2.15–2.00 (m, 1H), 1.70–1.53 (m, 1H), 1.45 (s, 9H), 0.95–0.85 (m, 9H), 0.10–0.05 (m, 6H). ^13^C-NMR (150 MHz, CDCl_3_): δ 155.73, 138.05, 128.89, 128.26, 128.19, 86.66, 79.92, 72.86, 69.62, 68.92, 49.46, 41.96, 33.00, 28.79, 26.11, 18.33, −4.29, −4.65. HRMS–ESI: [M + H]^+^ calcd. for C_24_H_42_NO_5_Si: 452.2832; found 452.2825. 

(**3b**) ${\left[\alpha \right]}_{D}^{20}$ –25 (*c* 1, CHCl_3_). FTIR (ATR): 2951, 2929, 1669, 1413, 1250, 1165, 1074, 836, 776 cm^−1^. ^1^H-NMR (400 MHz, CDCl_3_): δ 7.40–7.25 (m, 5H), 4.71 (d, *J* = 12.12 Hz, 1H), 4.62 (d, *J* = 12.08 Hz, 1H), 4.10–3.95 (m, 2H), 3.95–3.73 (m, 4H), 3.47–3.25 (m, 1H), 3.10–2.90 (m, 1H), 2.50–2.30 (m, 1H), 1.62 (s, broad, 1H), 1.47 (s, 9H), 0.91 (s, 9H), 0.08 (s, 6H). ^13^C-NMR (150 MHz, CDCl_3_): δ 158.32, 139.28, 128.64, 127.77, 127.67, 80.95, 78.03, 74.72, 73.07, 69.79, 54.87, 51.24, 35.16, 28.74, 26.15, 18.40, −4.36, −4.45. HRMS–ESI: [M + H]^+^ calcd. for C_24_H_42_NO_5_Si: 452.2832; found 452.2825.

#### 3.2.3. (3*R*,4*R*,5*S*)-4-(Benzyloxy)-3-((*tert*-butyldimethylsilyl)oxy)-5-hydroxyazepane (**(5*S*)-4a**)

Alcohol **3a** (5.0 mg, 11 µmol) was dissolved in trifluoroacetic acid (TFA, 500 µL) at 25 °C and stirred for 5 min before evaporation of the TFA under N_2_ flow. Traces of TFA were then removed under high-vacuum (0.01 torr, 25 °C) for 3 h, yielding alcohol **(5*S*)-4a** as a light yellow oil (3.9 mg, quant.). ${\left[\alpha \right]}_{D}^{20}$ +8 (*c* 0.12, CHCl_3_). FTIR (ATR): 2953, 2929, 1671, 1200, 1132, 835, 778 cm^−1^. ^1^H-NMR (400 MHz, CDCl_3_): δ 9.95 (s, broad, 1H), 8.45 (s, broad, 1H), 7.29–7.40 (m, 5H), 4.80 (d, *J* = 11.7 Hz, 1H), 4.57 (d, *J* = 11.6 Hz, 1H), 4.42 (dd, *J* = 7.1, 2.8 Hz, 1H), 4.01 (ddd, *J* = 6.8, 6.8, 4.4 Hz, 1H), 3.58 (dd, *J* = 6.8, 1.3 Hz, 1H), 3.32 (m, 1H), 3.29 (m, 2H), 3.28 (s, broad, 1H), 3.16 (m, 1H), 2.26 (dddd, *J* = 15.9, 8.1, 4.3, 3.6 Hz, 1H), 1.92 (dddd, *J* = 15.8, 9.0, 6.5, 2.6 Hz, 1H), 0.89 (s, 9H), 0.11 (s, 3H), 0.09 (s, 3H). ^13^C-NMR (150 MHz, CDCl_3_): δ 137.78, 128.98, 128.50, 128.23, 85.62, 73.97, 68.38, 67.41, 47.24, 41.39, 29.60, 25.99, 18.24, −4.50, −4.84. HRMS–ESI: [M]^+^ calcd. for C_19_H_34_NO_3_Si: 352.2308; found 352.2300.

#### 3.2.4. (3*R*,4*R*,6*S*)-4-(Benzyloxy)-3-((*tert*-butyldimethylsilyl)oxy)-5-hydroxyazepane (**(6*R*)-4b**)

Alcohol **3b** (5.0 mg, 11 µmol) was *N*-Boc deprotected as above for **3a**, yielding alcohol **(6*R*)-4b** as a pale yellow oil (3.9 mg, quant.). ${\left[\alpha \right]}_{D}^{20}$ +8 (*c* 0.07, CHCl_3_); FTIR (ATR): 2953, 2929, 1672, 1199, 1132, 835, 778 cm^−1^. ^1^H-NMR (400 MHz, CDCl_3_): δ 9.50 (s, broad, 1H), 8.42 (s, broad, 1H), 7.37–7.25 (m, 5H), 4.53 (d, *J* = 11.8 Hz, 1H), 4.48 (d, *J* = 11.8 Hz, 1H), 4.26 (m, 1H), 4.21 (m, 1H), 3.84 (dd, *J* = 9.5, 2.3 Hz, 1H), 3.41 (m, 2H), 3.33 (dd, *J =* 13.7, 3.3 Hz, 1H), 3.17 (dd, *J* = 13.3, 5.5 Hz, 1H), 2.26 (dq, *J* = 14.4, 4.8 Hz, 1H), 2.01 (dt, *J* = 14.5, 3.8 Hz, 1H), 0.89 (s, 9H), 0.08–0.06 (m, 6H). ^13^C-NMR (150 MHz, CDCl_3_): δ 138.37, 128.75, 128.06, 127.85, 77.48, 72.50, 71.48, 63.69, 50.63, 48.73, 34.63, 26.01, 18.26, −4.37, −4.94. HRMS–ESI: [M + H]^+^ calcd. for C_19_H_34_NO_3_Si: 352.2308; found 352.2300.

#### 3.2.5. *tert*-Butyl (3*S*,4*S*)-4-(benzyloxy)-3-((*tert*-butyldimethylsilyl)oxy)-5-oxo-azepine-1-carboxylate (**5a**); *tert*-Butyl (3*S*,4*S*)-4-(benzyloxy)-3-((*tert*-butyldimethylsilyl)oxy)-6-oxo-azepine-1-carboxylate (**5b**)

Pyridinium chlorochromate (PCC, 130 mg, 0.60 mmol) was added to a well-stirred suspension of alcohol diastereomers **3a** and **3b** (91 mg, 0.20 mmol) and 4 Å MS (137 mg) in dry DCM (10 mL) under N_2_ atmosphere at 25 °C. The reaction mixture was then stirred for 1 h followed by passage through a plug of dry 4 Å MS and flash column chromatography (petroleum ether/ethyl acetate 9:1) to yield pure fractions of **5a** (28.5 mg) and **5b** (40.2 mg) in 75% overall yield. 

(**5a**) ${\left[\alpha \right]}_{D}^{20}$ +11 (*c* 1, CHCl_3_); FTIR (ATR): 2955, 2929, 1696, 1406, 1159, 1093, 834, 776 cm^−1^. ^1^H-NMR (400 MHz, CDCl_3_): δ 7.40–7.25 (m, 5H), 4.73–4.66 (m, 2H), 4.15–4.05 (m, 1H), 3.95–3.60 (m, 4H), 3.45–3.35 (m, 1H), 2.85–2.55 (m, 2H), 1.43 (s, 9H), 0.91–0.87 (m, 9H), 0.14–0.06 (m, 6H). ^13^C-NMR (150 MHz, CDCl_3_): δ 209.71, 154.90, 137.92, 128.83, 128.82, 128.00, 90.98, 80.38, 74.23, 72.36, 50.51, 42.65, 41.84, 28.61, 26.06, 18.35, −4.56, −4.66. HRMS–ESI: [M+H]^+^ calcd. for C_24_H_40_NO_5_Si: 450.2676; found 450.2669.

(**5b**) ${\left[\alpha \right]}_{D}^{20}$ +32 (*c* 1, CHCl_3_); FTIR (ATR): 2954, 2929, 1697, 1409, 1155, 1109, 1079, 835, 775 cm^−1^. ^1^H-NMR (400 MHz, CDCl_3_): δ 7.40–7.20 (m, 5H), 4.80–4.60 (m, 2H), 4.60–4.20 (m, 1H), 4.10–3.70 (m, 3H), 3.60–3.10 (m, 2H), 2.81 (dd, *J* = 14.36, 8.16 Hz, 1H), 2.6 (dd, *J =* 21.09, 13.93 Hz, 1H), 1.50 (s, 5H), 1.43 (s, 4H), 0.93 (s, 9H), 0.20–0.08 (m, 6H). ^13^C-NMR (150 MHz, CDCl_3_): δ 208.93, 155.58, 138.42, 128.66, 127.95, 127.64, 81.28, 76.79, 74.55, 73.06, 59.13, 49.84, 43.74, 28.85, 26.12, 18.35, −4.34, −4.58. HRMS–ESI: [M + H]^+^ calcd. for C_24_H_40_NO_5_Si: 450.2676; found 450.2668.

#### 3.2.6. (3*S*,4*S*)-4-(Benzyloxy)-3-((*tert*-butyldimethylsilyl)oxy)-5-oxoazepan-1-ium (**6a**)

Ketone **6a** (5.0 mg, 11 µmol) was produced as above for **(5*S*)-4a** and was isolated as a pale light yellow powder (3.9 mg, 95%). ${\left[\alpha \right]}_{D}^{20}$ +10 (*c* 1, CHCl_3_); FTIR (ATR): 2953, 2930, 1672, 1171, 1132, 835, 781 cm^−1^. ^1^H-NMR (400 MHz, CDCl_3_): δ 7.38–7.26 (m, 5H), 4.84 (d, *J* = 11.68 Hz, 1H), 4.47 (d, *J* = 11.68 Hz, 1H), 4.32 (s, broad, 1H), 4.30 (m, 1H), 3.74–3.60 (m, 1 H), 3.50 (dd, *J* = 13.62, 5.98 Hz, 1H), 3.45–3.20 (m, 2H), 2.79 (t, *J* = 4.46, 2H), 0.85 (s, 9H), 0.09 (s, 3H), 0.08 (s, 3H). ^13^C-NMR (150 MHz, CDCl_3_): δ 202.10, 137.19, 128.93, 128.28, 128.09, 87.18, 69.93, 51.33, 41.99, 38.29, 25.93, 25.84, 18.24, −4.63, −4.95. HRMS–ESI: [M + H]^+^ calcd. for C_19_H_32_NO_3_Si: 350.2152; found 350.2142.

#### 3.2.7. (3*S*,4*S*)-4-(Benzyloxy)-3-((*tert*-butyldimethylsilyl)oxy)-6-oxoazepan-1-ium (**6b**)

Ketone **6b** (5.0 mg, 11 µmol) was produced as above for **(5*S*)-4a** and was isolated as a pale light yellow oil (3.9 mg, 95%). ${\left[\alpha \right]}_{D}^{20}$ +25 (*c* 1, CHCl_3_); FTIR (ATR): 2955, 2929, 1676, 1655, 1175, 1136, 836, 778 cm^−1^. ^1^H-NMR (400 MHz, CDCl_3_): δ 7.37–7.26 (m, 5H), 4.67 (s, broad, 2H), 4.32 (m, 1H), 3.84 (m, 1H), 3.68 (d, *J* = 17.92 Hz, 2H), 3.43 (dd, *J* = 13.20, 9.53 Hz, 1H), 3.33 (dd, *J* = 13.20, 3.99 Hz, 1H), 3.13 (dd, *J* = 15.26, 8.22 Hz, 1H), 3.03 (dd, *J* = 15.68, 0.60 Hz, 1H), 0.90 (s, 9H), 0.10 (s, 3H), 0.09 (s, 3H). ^13^C-NMR (150 MHz, CDCl_3_): δ 200.46, 137.45, 128.88, 128.42, 128.11, 75.48, 73.21, 71.68, 56.42, 49.13, 43.51, 25.92, 18.25, −4.64, −4.96. HRMS–ESI: [M + H]^+^ calcd. for C_19_H_32_NO_3_Si: 350.2152; found 350.2142.

#### 3.2.8. *tert*-Butyl (3*S*,4*R*)-4-(benzyloxy)-3-((*tert*-butyldimethylsilyl)oxy)azepane-1-carboxylate (**3e**)

To a solution of tetrahydroazepine **2** (75 mg, 170 µmol) in methanol (350 µL) was added 5% *w*/*w* Pd/C (2 mg, 1 µmol, 0.5 mol %). The suspension was then stirred under H_2_ (1 atm) for 16 h until completion as confirmed by TLC. Catalyst was removed by passage through a plug of Celite with methanol rinsing. Solvent was then removed in vacuo to yield azepane **9** as a colourless oil (36 mg, 48%). ${\left[\alpha \right]}_{D}^{20}$ −200 (*c* 0.14, CHCl_3_); FTIR (ATR): 2929, 2856, 1691, 1165, 1082, 834, 774 cm^−1^. ^1^H-NMR (400 MHz, CDCl_3_): 7.40–7.23 (m, 5H), 4.70–4.55 (m, 2H), 3.93 (m, 0.5H), 3.87–3.54 (m, 3.5H), 3.20–3.00 (m, 2H), 2.05–1.94 (m, 1H), 1.85–1.69 (m, 1.5H), 1.68–1.57 (m, 1H), 1.56–1.38 (m, 9.5H), 0.95–0.88 (m, 9H), 0.13–0.05 (m, 6H). δ ^13^C-NMR (150 MHz, CDCl_3_): δ 155.57, 139.54, 128.57, 127.65, 127.58, 80.39, 74.51, 72.30, 72.19, 50.06, 46.76, 28.89, 26.20, 26.20, 23.43, 18.44, −4.26, −4.38. HRMS–ESI: [M + H]^+^ calcd. for C_24_H_42_NO_4_Si: 436.2883; found 436.2875.

#### 3.2.9. (3*S*,4*R*)-4-(Benzyloxy)-3-((*tert*-butyldimethylsilyl)oxy)azepan-1-ium (**4c**)

Azepane **3e** (5.0 mg, 11 µmol) was *N*-Boc deprotected as above for **3a**, yielding **4c** as a colourless oil (3.9 mg, quant.). ${\left[\alpha \right]}_{D}^{20}$ −84 (*c* 0.03, CHCl_3_); FTIR (ATR): 2930, 2857, 1674, 1199, 1131, 834, 778 cm^−1^. ^1^H-NMR (400 MHz, CDCl_3_): δ 10.12 (s, broad, 1H), 8.07 (s, broad, 1H), 7.40–7.26 (m, 5H), 4.67–4.51 (m, 2H), 4.22 (m, 1H), 3.66–3.52 (m, 1H), 3.32 (m, 2H), 3.16 (m, 2H), 1.98 (m, 2H), 1.80 (m, 2H), 0.89 (s, 9H), 0.086 (m, 6H). ^13^C-NMR (150 MHz, CDCl_3_): δ 138.32, 128.79, 128.14, 127.94, 81.05, 72.42, 71.06, 47.28, 45.94, 26.71, 26.03, 20.97, 18.32, −4.41, −4.84. HRMS–ESI: [M + H]^+^ calcd. for C_19_H_34_NO_2_Si: 336.2359; found 336.2348.

## 4. Conclusions

Overall, we have shown here that the late-stage oxidation of tetrahydroazepine **2** constitutes an efficient way to access novel oxo-azepines. Hydroboration of **2** with BH_3_∙SMe_2_ yielded azepanols **3a** and **3b** in a 3:2 product ratio and, following oxidation, afforded regioisomeric ketones **5a** and **5b**. Separation of these ketones was readily achieved to provide **5a** and **5b** in 29% and 19% overall yield, respectively. Regioselectivity can be improved by using additives such as Rh(COD)(DPPB)BF_4_, albeit with competitive hydrogenation (Table 3, Entry 4), to access ketones **5a** and **5b** in a 1:7 product ratio, over 8 steps and in 30% overall yield for **5b**. Azepanols **3a/b** and oxo-azepines **5a/b** are versatile synthons and, following complete deprotection, useful epitopes in the construction of novel azepane-based biological agents. As a point of comparison, the work of Spiegel et al. highlighted the synthetic utility of their C6 ketone **vi** in the concise synthesis of glucosepane via a sigmatropic rearrangement and cyclization sequence in a total of 13 steps and 12% overall yield from diacetone-d-glucose (Scheme 1b). Ketones **5a** and **5b** structurally resemble compound **vi** as trisubstituted oxo-azepines bearing a protected vicinal dihydroxyl motif with *syn* stereochemistry at C3 and C4. The availability of a more-efficient route may assist in future biological investigations focused on glucosepanes.

## Figures and Tables

**Table 1:** Screening of different hydroboration reagents.

Entry	Conditions	Conversion (%)	Ratio ^c^		
			3a	3b	3e
1	BH_3_∙SMe_2_, 25 °C, 18 h	99	58	42	0
2	BH_3_∙THF, 25 °C, 18 h	4.3 ^b^	69	31	0
3	BH_3_∙NMe_3_, 66 °C, 18 h	0 ^b^	0	0	0
4	CatBH, 66 °C, 18 h	0 ^b^	0	0	0
5 ^a^	CatBH, 66 °C, 18 h	100	0	0	100
6	9-BBN, 66 °C, 18 h	0 ^b^	0	0	0
7	PinBH, 25 °C, 18 h	0 ^b^	0	0	0

^a^ 10 mol % Wilkinson’s catalyst was added. ^b^ Starting material was recovered. ^c^ All reactions were carried out in THF with product ratios determined by HPLC. Formation of **3c** and **3d** was not observed.

**Table 2:** Catalyst screen for the hydroboration of **2**.

Entry	Conditions	Conversion (%)	Ratio ^b^		
			3a	3b	3e
1	CatBH, 5 mol % Ir(COD)(PCy_3_)(Py)PF_6_, THF, 66 °C, 18 h	44 ^a^	0	0	100
2	PinBH, 5 mol % Rh(COD)(DPPB)BF_4_, DCE, 25 °C, 18 h	75 ^a^	5	34	61
3	PinBH, 5 mol % Rh(COD)(DPPB)BF_4_, 2 mol % FAB, DCE, 66 °C, 18 h	58 ^a^	2	8	90
4	PinBH, 3 mol % CuCl, 3.3 mol % TangPhos, 6 mol % NaO^t^Bu, toluene, 66 °C, 18 h	0 ^a^	0	0	0
5	PinBH, 3 mol % CuCl, 3.3 mol % (*R*)-DTBM SegPhos, 6 mol % NaO^t^Bu, toluene, 66 °C, 18 h	0 ^a^	0	0	0

^a^ Starting material was recovered. ^b^ No. **3c** or **3d** was formed in these reactions.

**Table 3:** Solvent screen for the hydroboration of **2** using PinBH and 5–10 mol % Rh(COD)(DPPB)BF_4_.

Entry	Conditions	Conversion (%)	Ratio ^b^		
			3a	3b	3e
1	DCE, 60 °C, 18 h	70	7	54	39
2 ^a^	DCE, 60 °C, 18 h	37	3	14	83
3	THF, 25 °C, 18 h	99	8	48	44
4	THF, 60 °C, 18 h	99	8	58	34
5	Toluene, 25 °C, 18 h	98	5	39	56
6	Toluene, 60 °C, 18 h	100	6	44	50
7 ^a^	Toluene, 60 °C, 18 h	44	11	57	32

^a^ 1 mol % catalyst loading. ^b^ No **3c** or **3d** was formed in these reactions.

## Supplementary Materials

Supplementary materials are available online. 2D-NMR spectral data for compounds **4a/b**, **6a/b** and **4c**; LC–MS data for key metal-catalyzed hydroboration reactions.
