# Supplementary material for: Synthesis of Substituted Oxo-Azepines by Regio- and Diastereoselective Hydroxylation
Source: Molecules. 2017 Oct 31;22(11):1871. doi: 10.3390/molecules22111871 (PMC6150284; doi:10.3390/molecules22111871)

Supplementary Information

Synthesis of Substituted Oxo-azepines by Regio- and Diastereo-selective Hydroxylation

Harold Spedding, Peter Karuso, Fei Liu*

Department of Molecular Sciences, Macquarie University, Sydney, NSW 2109, Australia

Corresponding Author – Fax: +61-2-9850-8313; E-mail: fei.liu@mq.edu.au

Contents

Table S1. Structural assignment summary for azepanol **(5*S*)-4a** 3

^{1}H-^{13}C HSQC **(5*S*)-4a** 4

^{1}H-^{13}C HMBC **(5*S*)-4a** 4

^{1}H-^{1}H COSY **(5*S*)-4a** 5

^{1}H-^{1}H NOESY **(5*S*)-4a** 5

Table S2. Structural assignment summary for azepanol (5R)-4a characterized as a mixture with **(5*S*)-4a** 6

^{1}H-^{13}C HSQC **(5*R*)-4a** 7

^{1}H-^{13}C HMBC **(5*R*)-4a** 7

^{1}H-^{1}H COSY **(5*R*)-4a** 8

^{1}H-^{1}H ROESY **(5*R*)-4a** 8

Table S3. Structural assignment summary for azepanol **(6*R*)-4b** 9

^{1}H-^{13}C HSQC **(6*R*)-4b** 10

^{1}H-^{13}C HMBC **(6*R*)-4b** 10

^{1}H-^{1}H COSY **(6*R*)-4b** 11

^{1}H-^{1}H NOESY **(6*R*)-4b** 11

Table S4. Structural assignment summary for azepanol (6S)-4b characterized as a mixture with **(6*R*)-4b** 12

^{1}H-^{13}C HSQC **(6*S*)-4b** 13

^{1}H-^{13}C HMBC **(6*S*)-4b** 13

^{1}H-^{1}H COSY **(6*S*)-4b** 14

^{1}H-^{1}H ROESY **(6*S*)-4b** 14

Table S5. Structural assignment summary for hydrogenation product **4c** 15

^{1}H-^{13}C HSQC **4c** 16

^{1}H-^{13}C HMBC **4c** 16

^{1}H-^{1}H COSY **4c** 17

^{1}H-^{1}H NOESY **4c** 17

Table S6. Structural assignment summary for 5-Oxoazepane **6a** 18

^{1}H-^{13}C HSQC **6a** 19

^{1}H-^{13}C HMBC **6a** 19

^{1}H-^{1}H COSY **6a** 20

^{1}H-^{1}H NOESY **6a** 20

Table S7. Structural assignment summary for 5-Oxoazepane **6b** 21

^{1}H-^{13}C HSQC **6b** 22

^{1}H-^{13}C HMBC **6b** 22

^{1}H-^{1}H COSY **6b** 23

^{1}H-^{1}H NOESY **6b** 23

LC-MS Traces of Key Hydroboration Catalyst Screen Reactions 24

Comparative ^{1}H NMR Spectra of Hydrogenation Product **3e** Versus Crude Reaction Mixtures 27

Structural Assignment of (5*S*)-4a

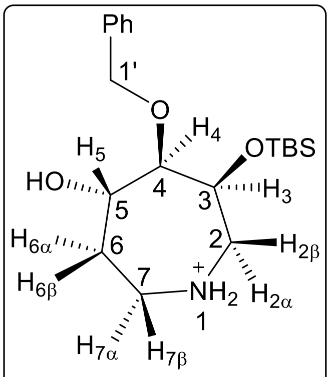

(5*S*)-4a

Table S1. Structural assignment summary for azepanol **(5*S*)-4a**

Pos.	δH, mult. (J in Hz)	δC	HMBC	COSY	NOESY
1′	4.57, d (11.6); 4.80, d (11.7)	74.0	4	-	3, 4, TBS
2α	3.16, m	47.2	-	2β, 3	2β, 3, 4, TBS
2β	3.32, m		-	2α, 3	2α, 3, TBS
3	4.42, dd (7.1, 2.8)	67.4	4, 5	2α, 2β	1′, 2α, 2β, 4, TBS
4	3.58, dd (6.8, 1.3)	85.6	1′, 2, 5, 6	5	1′, 2α, 3, 5, 6α, TBS
5	4.01, ddd (6.8, 6.8, 4.4)	68.4	3, 4, 7	4, 6α, 6β	4, 6α, 6β, 7α/β, TBS
6α	1.92, dddd (15.8, 9.0, 6.5, 2.6)	29.6	4, 5, 7	5, 6β, 7α/β	4, 5, 6β, 7α/β
6β	2.26, dddd (15.9, 8.1, 4.3, 3.6)		4, 5, 7	5, 6α, 7α/β	5, 6α, 7α/β
7α/β	3.29, m	41.4	-	6α, 6β	5, 6α, 6β
Ph	7.29-7.40, m	128.2, 128.5, 129.0, 137.8	1′	-	1′
TBS	0.09, s; 0.11, s; 0.89, s	−4.8, −4.5, 18.2, 26.0	TBS	-	1′, 2/β, 3, 4, 5, 6β, TBS

^{1}H-^{13}C HSQC **(5*S*)-4a**

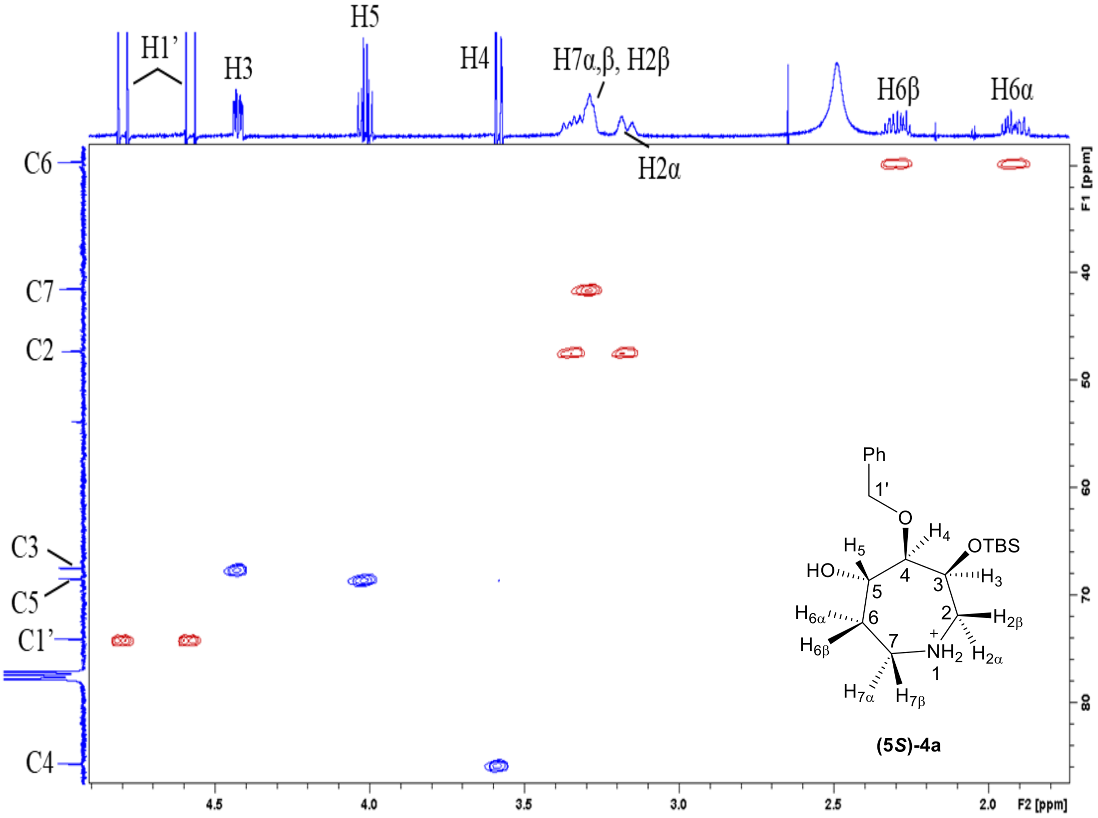

^{1}H-^{13}C HMBC **(5*S*)-4a**

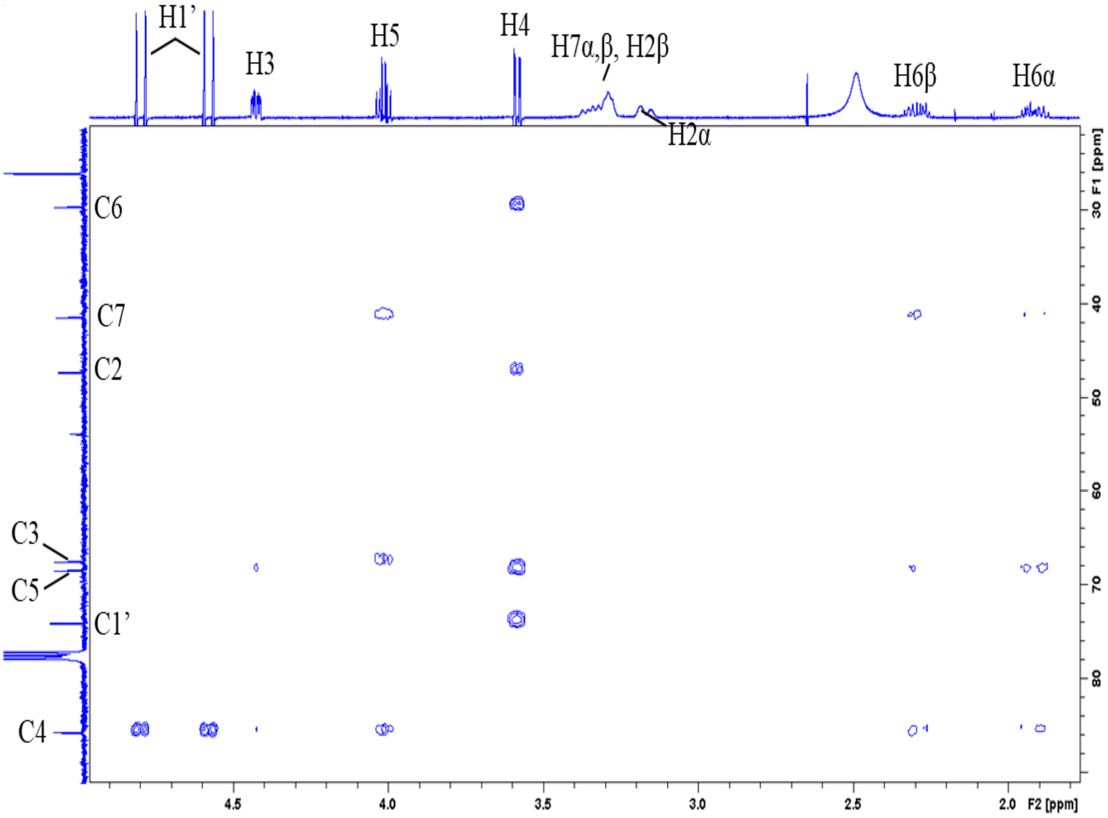

^{1}H-^{1}H COSY **(5*S*)-4a**

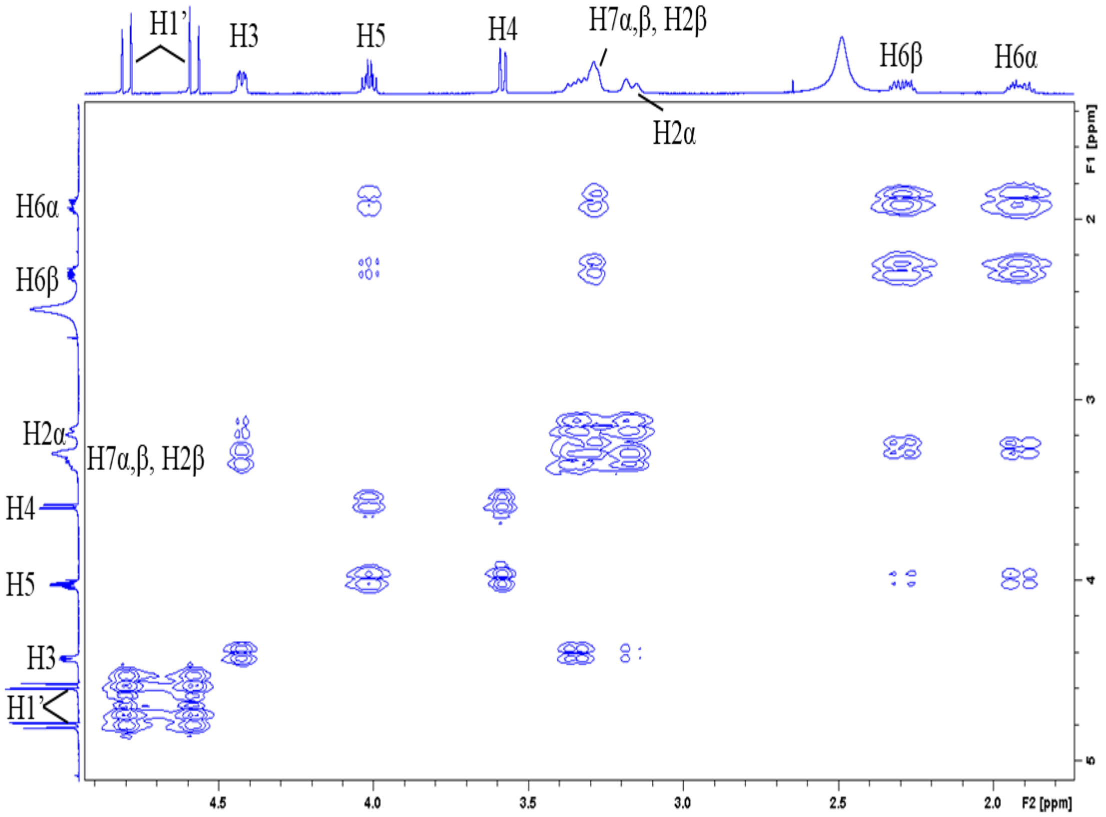

^{1}H-^{1}H NOESY **(5*S*)-4a**

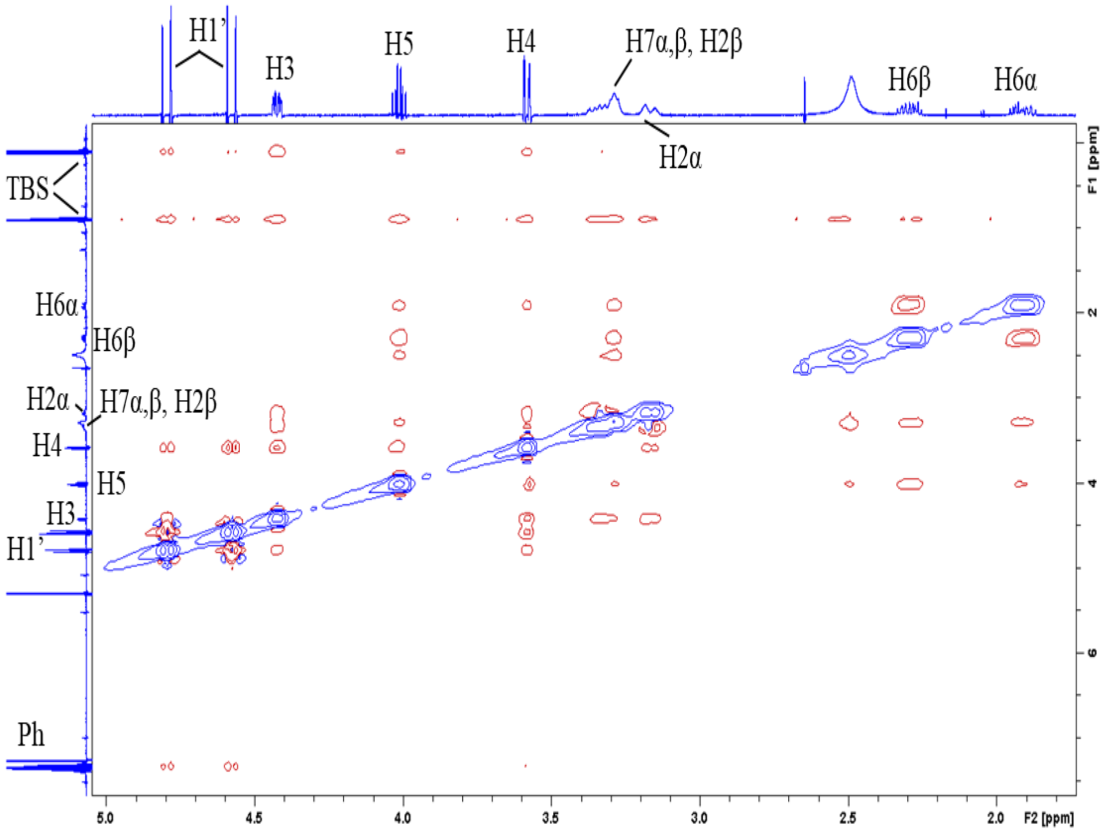

Structural Assignment of (5*R*)-4a

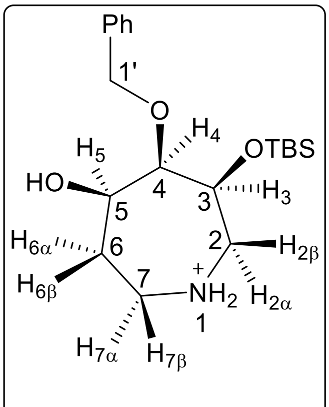

(5*R*)-4a

Table S2. Structural assignment summary for azepanol **(5*R*)-4a** characterized as a mixture with **(5*S*)-4a**

Pos.	δH, mult. (J in Hz)	δC	HMBC	COSY	NOESY
1′	4.68, d (11.6); 4.80, d (11.6)	73.9	4	-	4, TBS
2α	3.18, m	46.4	4	2β, 3	2β, 3, 7β, TBS
2β	3.43, m			2α, 3, 7α	2α, 3, 7α, TBS
3	4.28, m	69.5	5	2α, 2β, 4	2α, 7α, TBS
4	3.62, t (2.45)	83.4	1′, 2, 5, 6	3, 5	1′, 2α, 3, 5, 7α, TBS
5	4.17, dt (8.05, 2.85)	69.0	3, 7	4, 6α, 6β	2α, 2β, 4, 7α
6α	1.98, m	27.8	-	5, 6β, 7α, 7β	2α, 6β, 5, 7α
6β	2.19, m			5, 6α, 7α, 7β	2α, 2β, 3, 6α, 7α
7α	3.20, m	41.6	-	6α, 6β, 7β	3, 6α, 6β, 7β, TBS
7β	3.51, m			6α, 6β, 7α	6α, 6β, 7α, TBS
Ph	7.30-7.40, m	128.4, 128.7, 129.0, 137.5	1′	-	1′
TBS	0.08, s; 0.11, s; 0.90, s	−4.9, −4.6, 18.3, 30.0	TBS	-	1′, 2α, 2β, 3, 4, 7α, 7β

^{1}H-^{13}C HSQC **(5*R*)-4a**

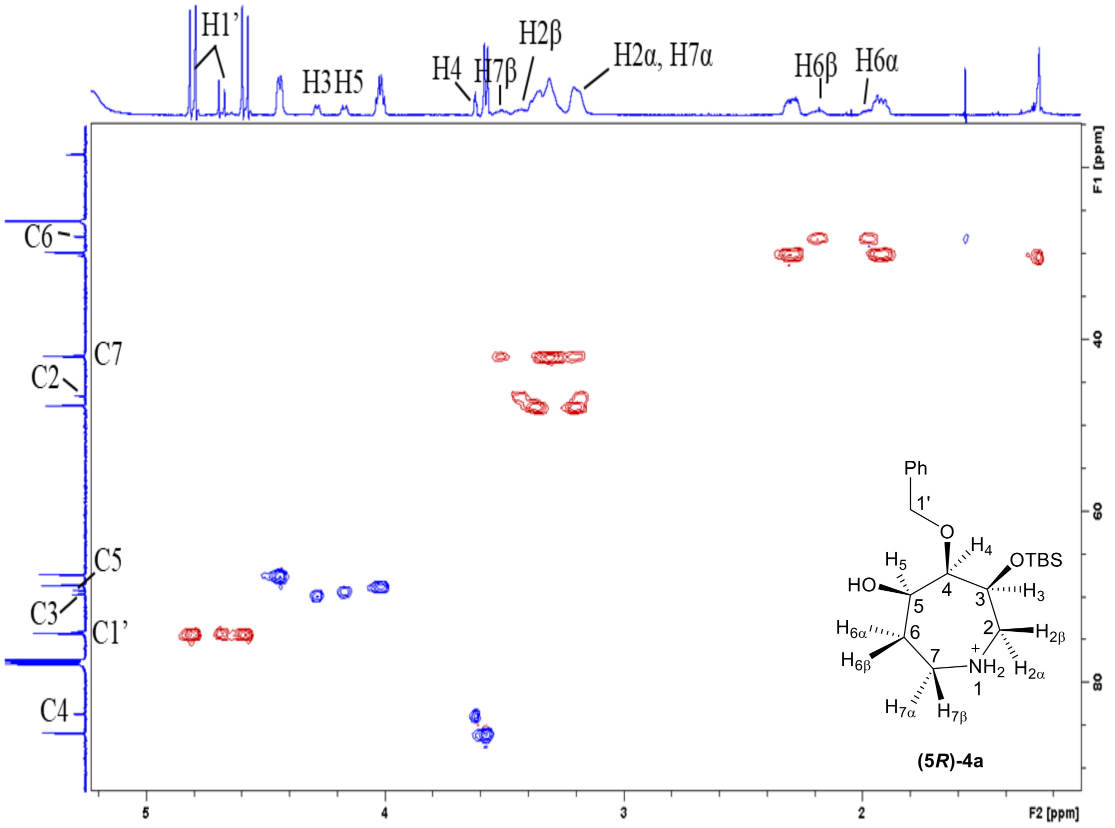

^{1}H-^{13}C HMBC **(5*R*)-4a**

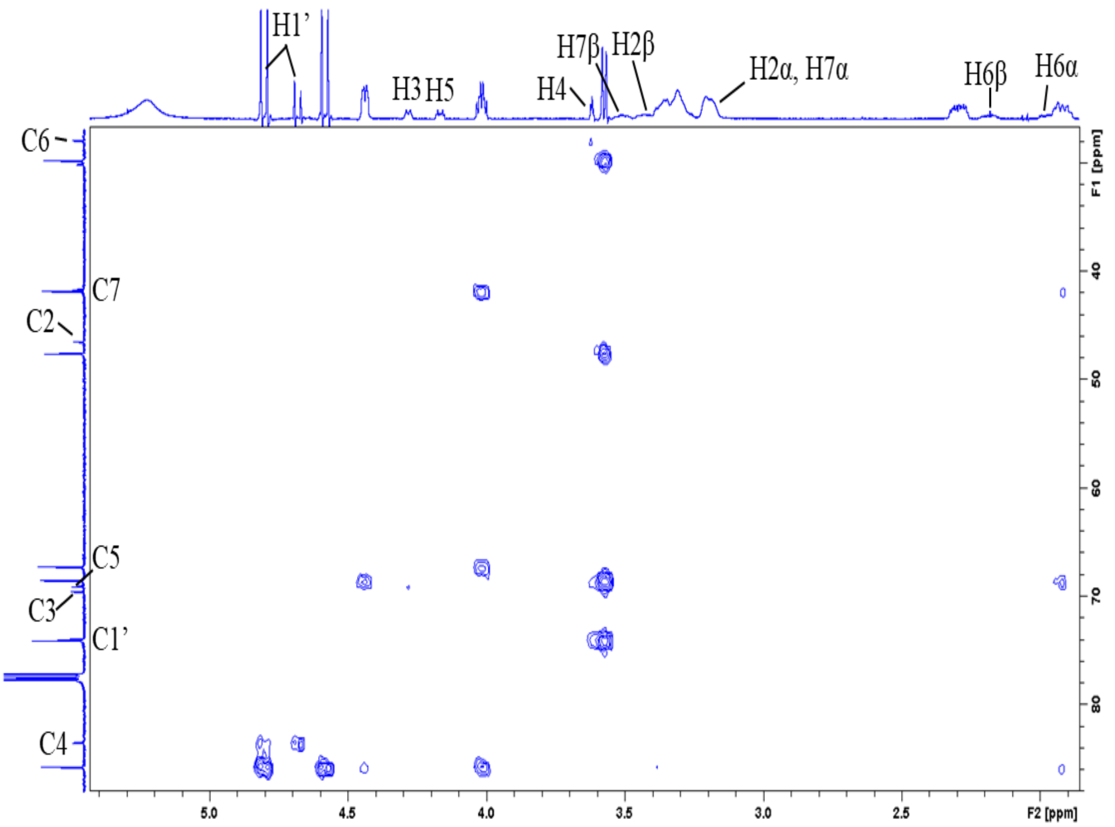

^{1}H-^{1}H COSY **(5*R*)-4a**

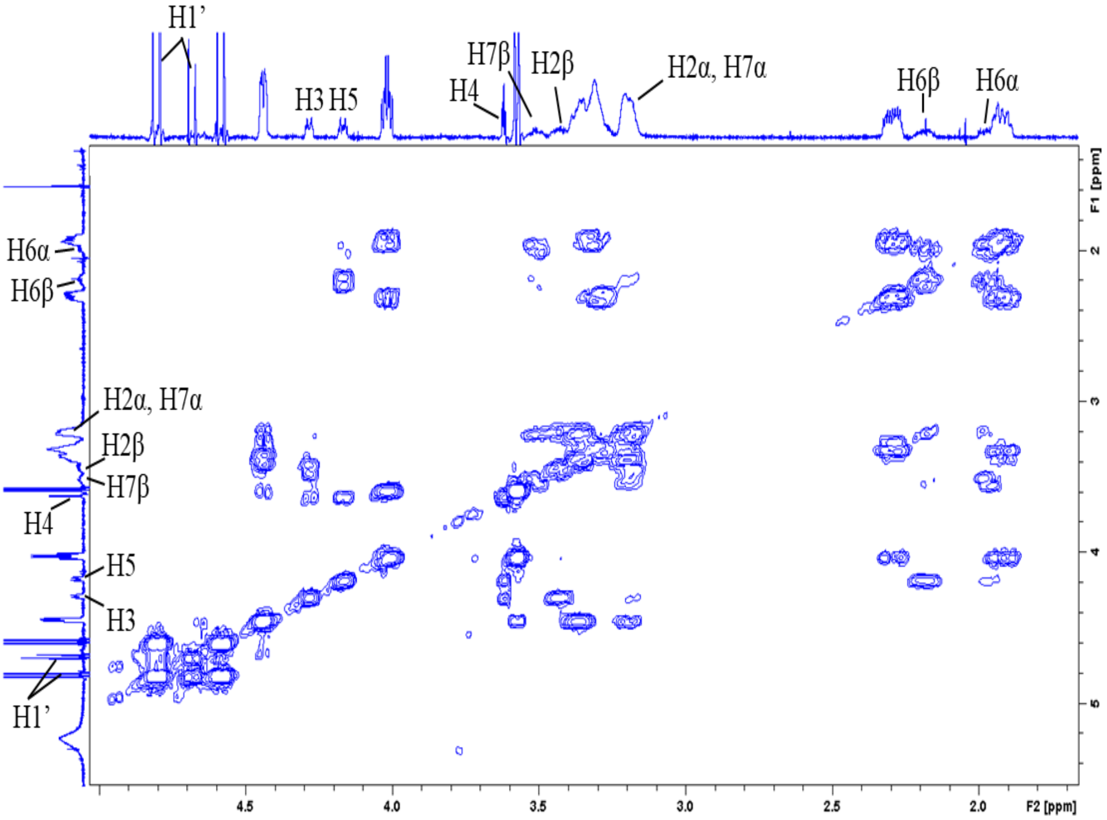

[1]H-^{1}H ROESY **(5*R*)-4a**

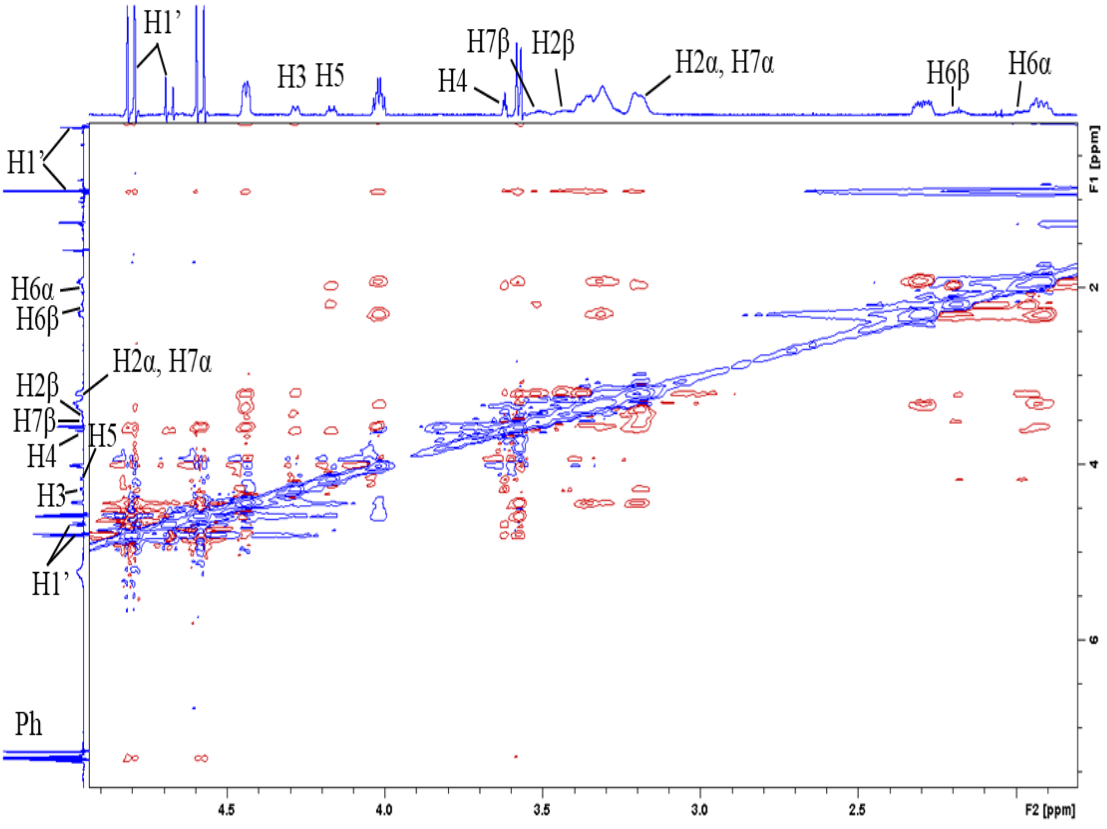

Structural Assignment of (6*R*)-4b

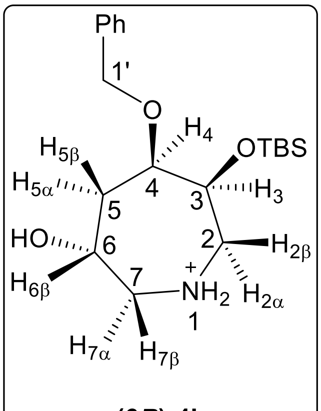

Table S3. Structural assignment summary for azepanol **(6*R*)-4b**

Pos.	δH, mult. (J in Hz)	δC	HMBC	COSY	NOESY
1′	4.48, d (11.8); 4.53, d (11.8)	72.5	4	-	3, 4, 5α, TBS
2α	3.33, dd (13.7, 3.3)	48.7	4	2β, 3	2β, 3, 4, TBS
2β	3.17, dd (13.3, 5.5)		3, 4	2α, 3	2α, 7
3	4.26, m	71.5	4, 5	2α, 2β	1′, 2α, 2β, 4, 5α, TBS
4	3.84, dd (9.5, 2.3)	77.5	1′, 5, 6	5α, 5β	1′, 2α, 3, 5α, 5β, TBS
5α	2.01, dt (14.5, 3.8)	34.6	3, 6	4, 5β, 6	1′, 3, 4, 5β, 6
5β	2.26, dq (14.4, 4.8)		3, 4, 6, 7	4, 5α, 6	4, 5α, 6, TBS
6	4.21, m	63.7	-	5α, 5β, 7α/β	5α, 5β, 7α/β
7α/β	3.41, m	50.6	-	6	2β, 6, TBS
Ph	7.25-7.37, m	127.8, 128.1, 128.7, 138.4	1′	-	1′, 3, 4
TBS	0.07, s; 0.08, s; 0.89, s	−4.9, −4.4, 18.3, 26.0	TBS	-	1′, 3, 4, 5β, 7α/β

^{1}H-^{13}C HSQC **(6*R*)-4b**

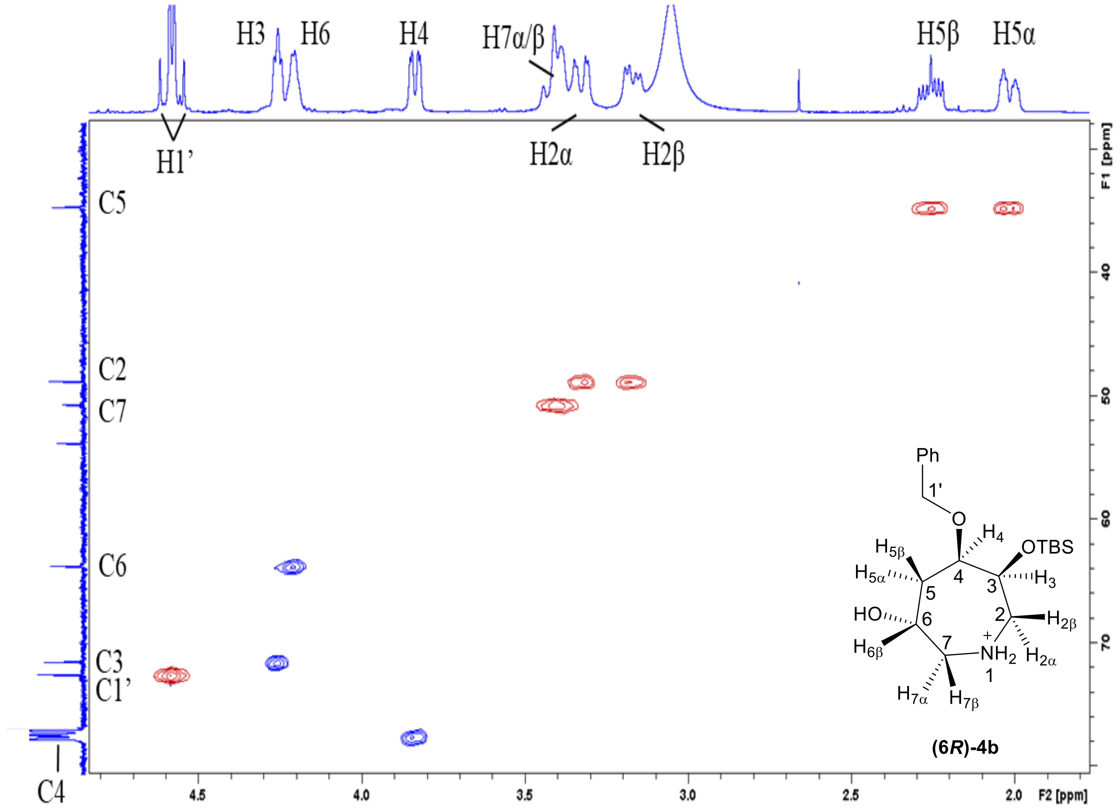

^{1}H-^{13}C HMBC **(6*R*)-4b**

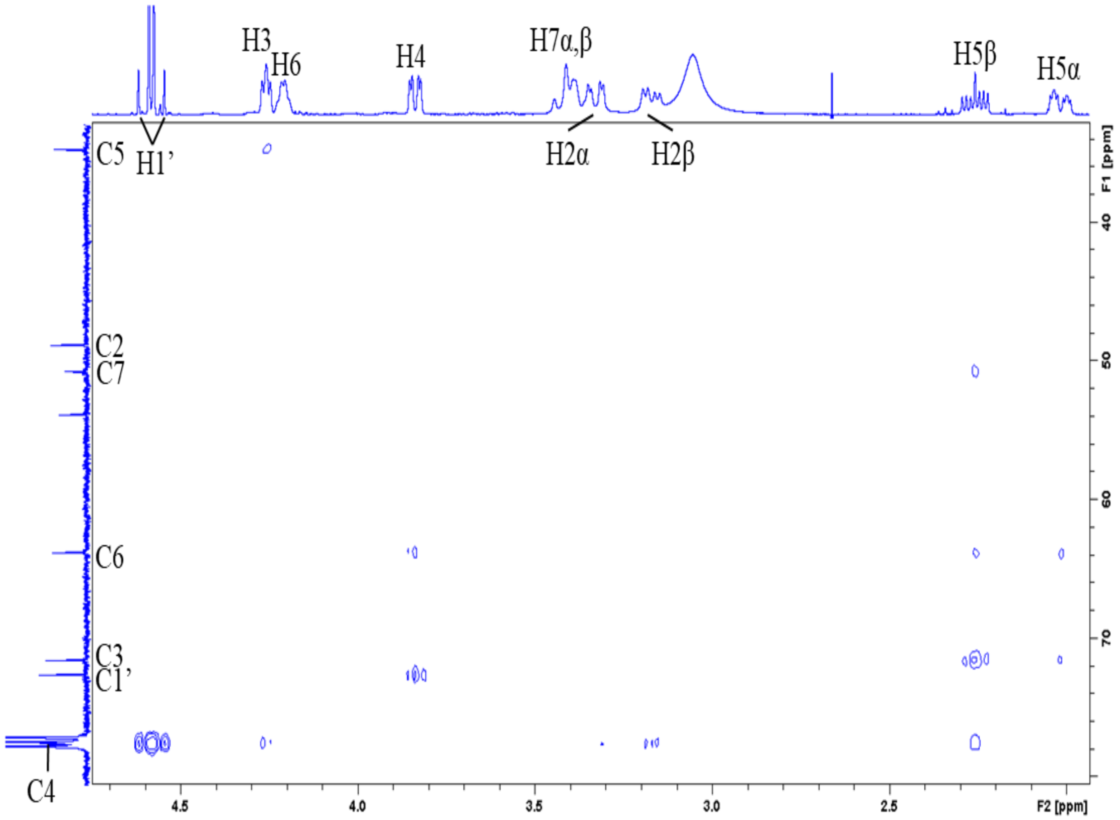

^{1}H-^{1}H COSY **(6*R*)-4b**

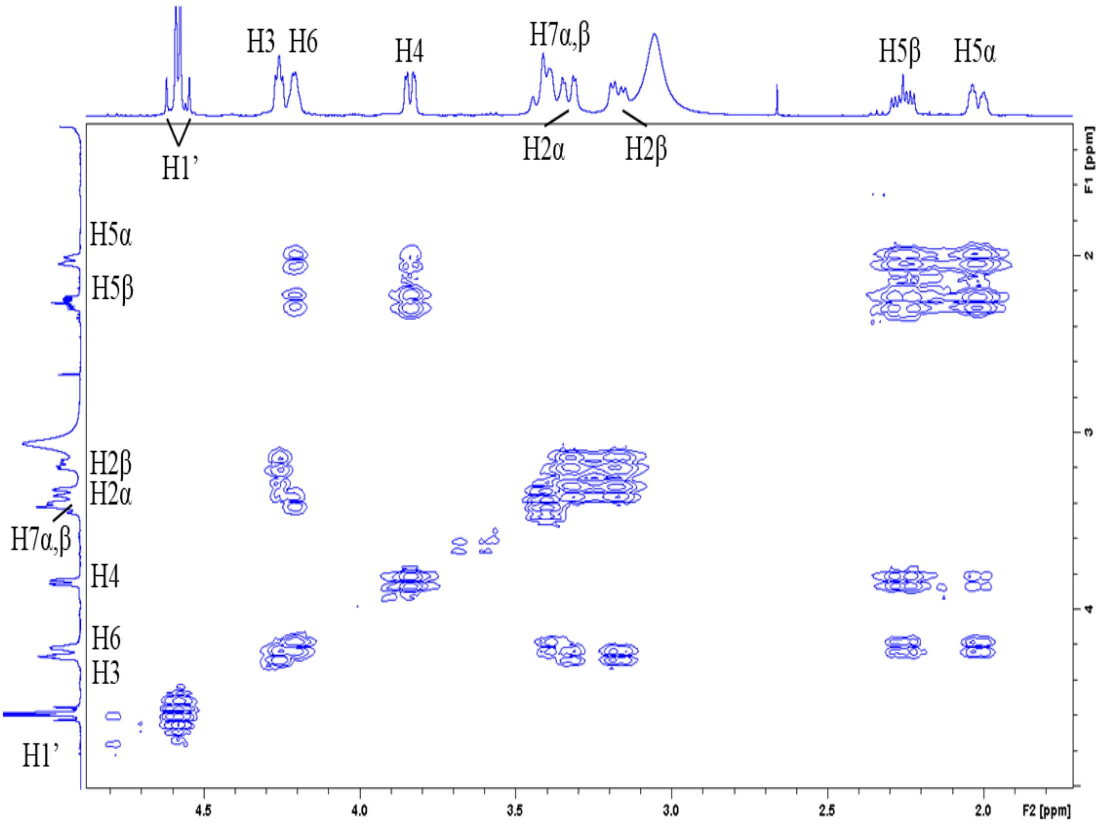

^{1}H-^{1}H NOESY **(6*R*)-4b**

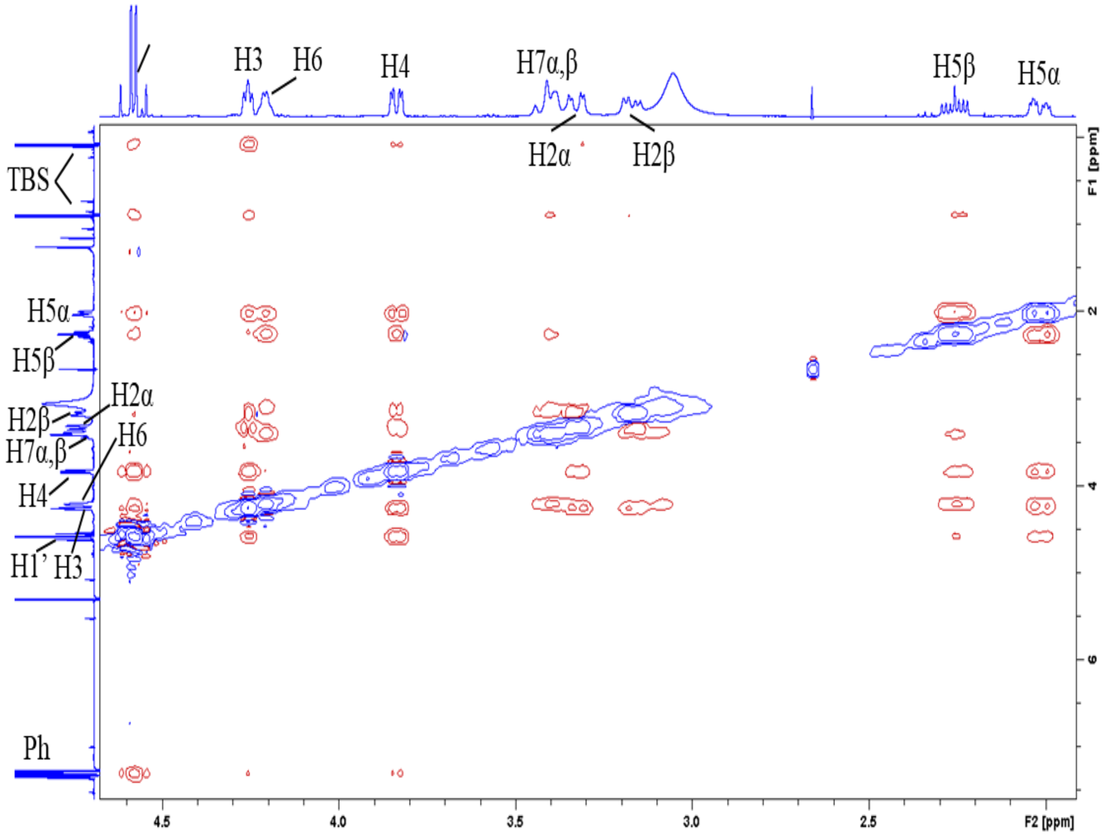

Structural Assignment of (6*S*)-4b

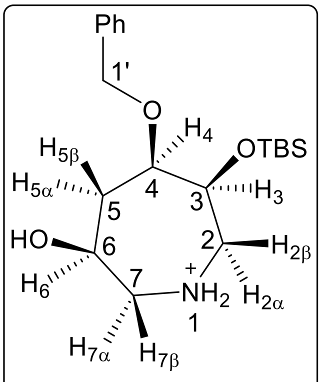

(6*S*)-4b

Table S4. Structural assignment summary for azepanol **(6*S*)-4b** characterized as a mixture with **(6*R*)-4b**

**Pos.**	**δH, mult. (J in Hz)**	**δC**	**HMBC**	**COSY**	**NOESY**
1′	4.66, d (12.0); 4.69, d (12.0)	72.8	4	-	3, 4, 5α, 5β, TBS
2α	3.25, dd (13.3, 4.4)	49.3	3, 4, 7	2β, 3	2β, 3, 4
2β	3.33, dd (13.3, 7.4)			2α, 3	2α, 3, 6, TBS
3	4.18, dd (7.2, 4.6)	71.1	2, 5	2α, 2β	1′, 4, 5α, 7α, TBS
4	3.52, dd (13.8, 4.4)	80.1	1′, 2, 5, 6	3, 5α, 5β	1′, 2α, 3, 5α, 5β, 7α, TBS
5α	1.97, ddd (15.3, 5.1, 2.9)	35.1	3, 4, 6, 7	4, 5β, 6	1′, 3, 4, 5β, 6
5β	2.34, ddd (15.2, 7.4, 4.1)			4, 5α, 6	1′, 4, 5α, 6
6	4.24, m	64.8	-	5α, 5β, 7β	4, 5α, 5β, 7α, 7β, TBS
7α	3.06, dd (14.1, 1.7)	51.2	2, 5, 6	6, 7α	2α, 3, 4, 5α, 6, 7β
7β	3.52, dd (13.7, 4.4)			6, 7β	6, 7α
Ph	7.20-7.40, m	127.8, 128.1, 129.0, 137.7	1′	-	1′
TBS	0.07, s; 0.08, s; 0.89, s	−4.4, −4.8, 18.3, 26.0	TBS	-	1′, 2β, 3, 4, 6

^{1}H-^{13}C HSQC **(6*S*)-4b**

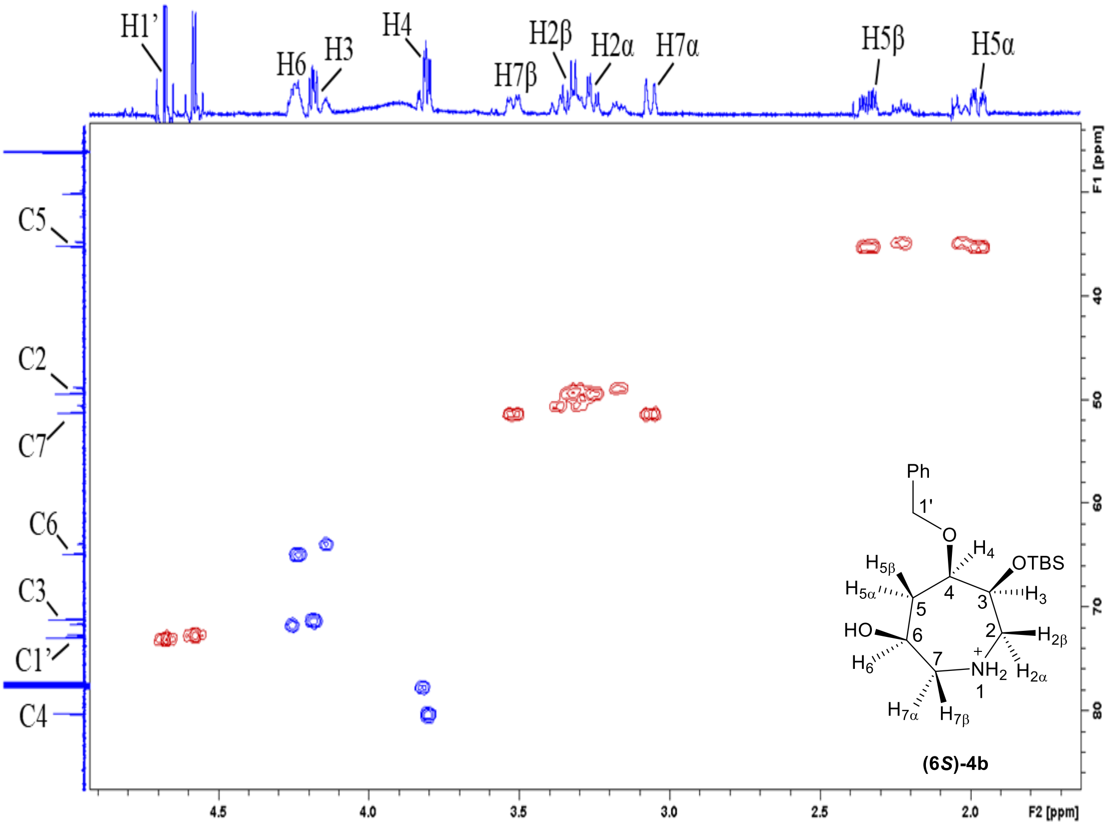

^{1}H-^{13}C HMBC **(6S)-4b**

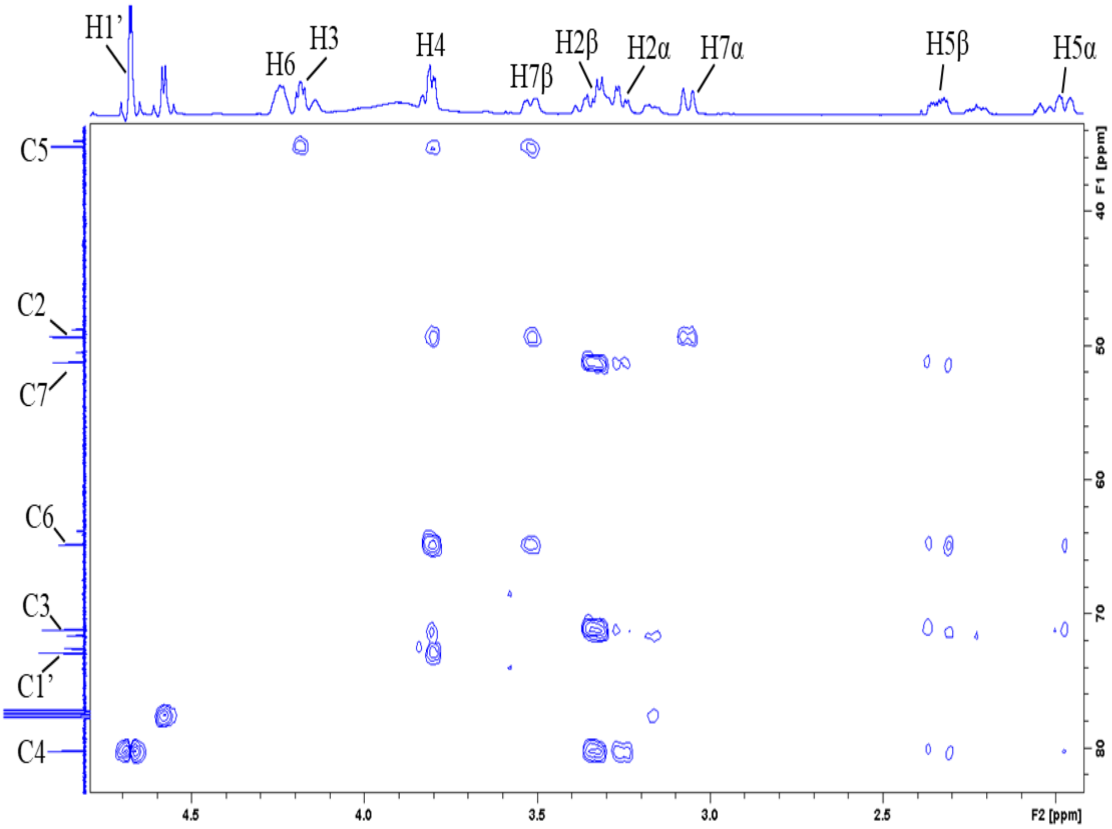

^{1}H-^{1}H COSY **(6*S*)-4b**

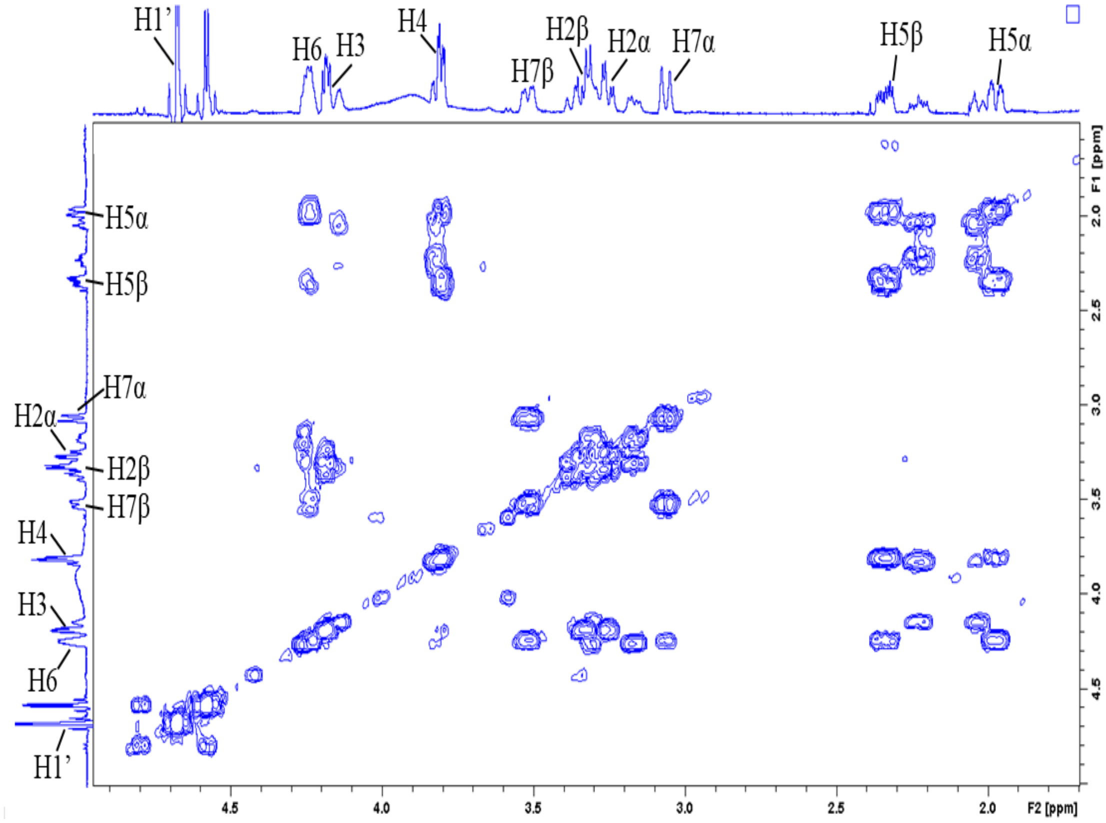

^{1}H-^{1}H ROESY **(6*S*)-4b**

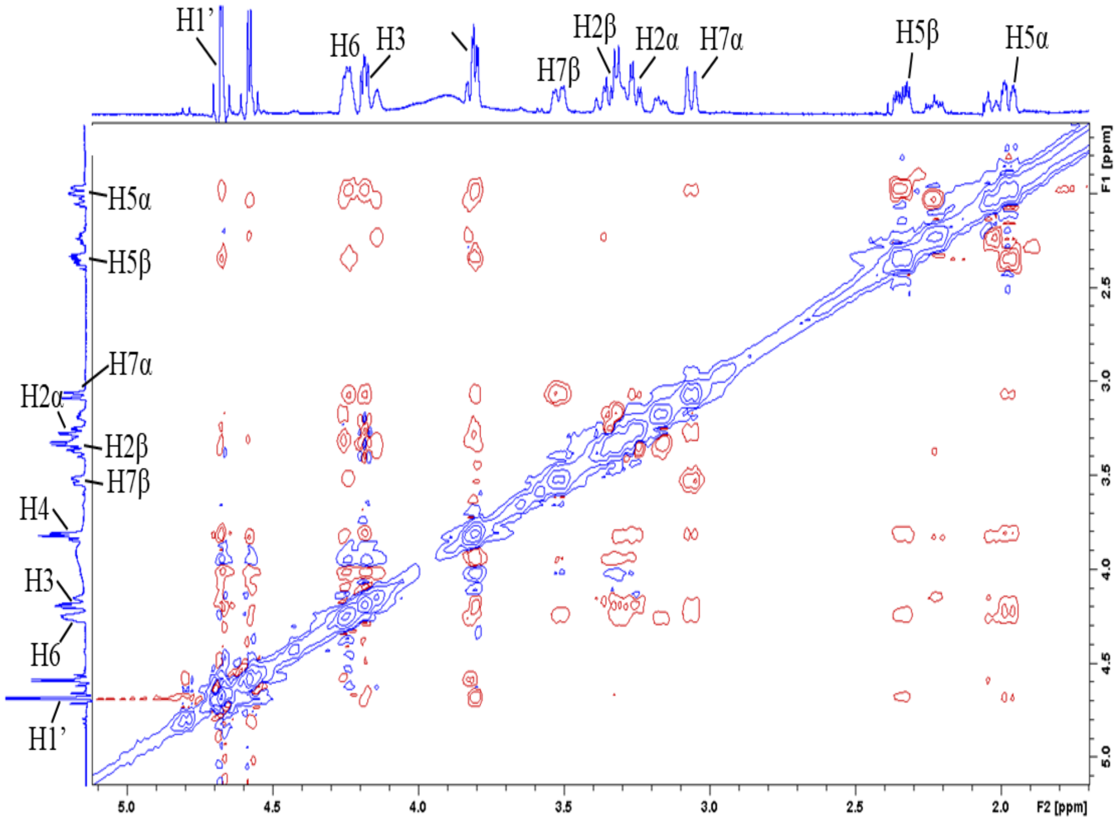

Structural Assignment of 4c

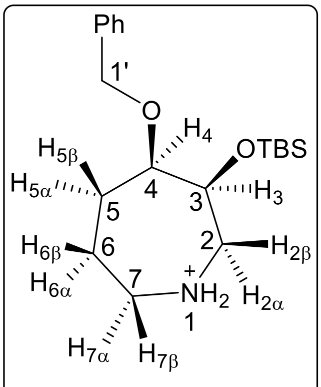

4c

Table S5. Structural assignment summary for hydrogenation product **4c**

Position	δ¹H /ppm	δ¹³C /ppm	HMBC	COSY	NOESY
1′	4.62, m	72.4	4	-	3, 4, 5α/β
2α	3.17, m	47.3	-	3, 2β, 7α/β	2β, 3
2β	3.33, m		-	3, 2α, 7α/β	2α, 3
3	4.22, m	71.1	5	2α, 2β, 4	5α
4	3.63, m	81.0	1′, 2, 6	3, 5α/β	1′, 2α, 3, 5α/β
5α	1.81, m	26.7	3, 4, 7	4, 5β, 6α/β	4, 5β
5β	1.98, m		4, 6, 7	4, 5α, 6α/β	4, 5α
6α	1.79, m	21.0	4, 7	5α/β, 6β, 7α/β	6β
6β	1.99, m		4, 7	5α/β, 6α, 7α/β	6α, 7α
7α	3.19, m	46.0	-	2α/β, 6α/β, 7β	7β
7β	3.35, m		-	2α/β, 6α/β, 7α	7α

^{1}H-^{13}C HSQC **4c**

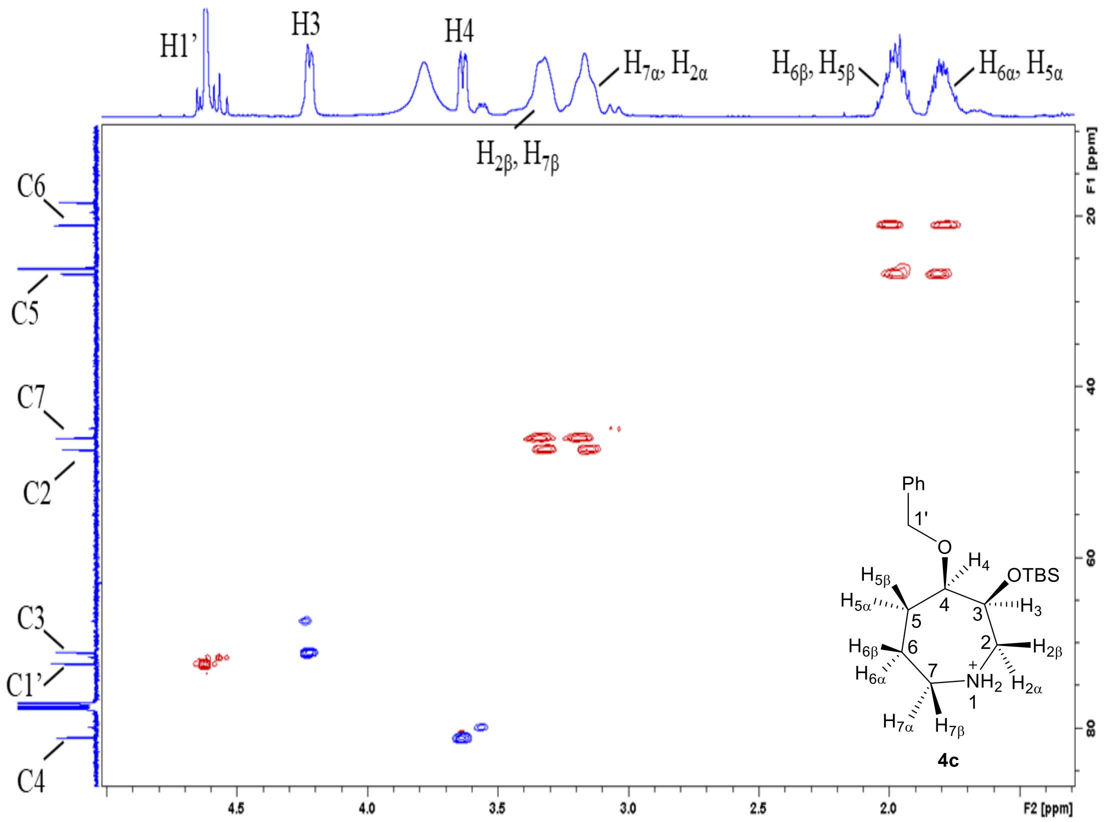

^{1}H-^{13}C HMBC **4c**

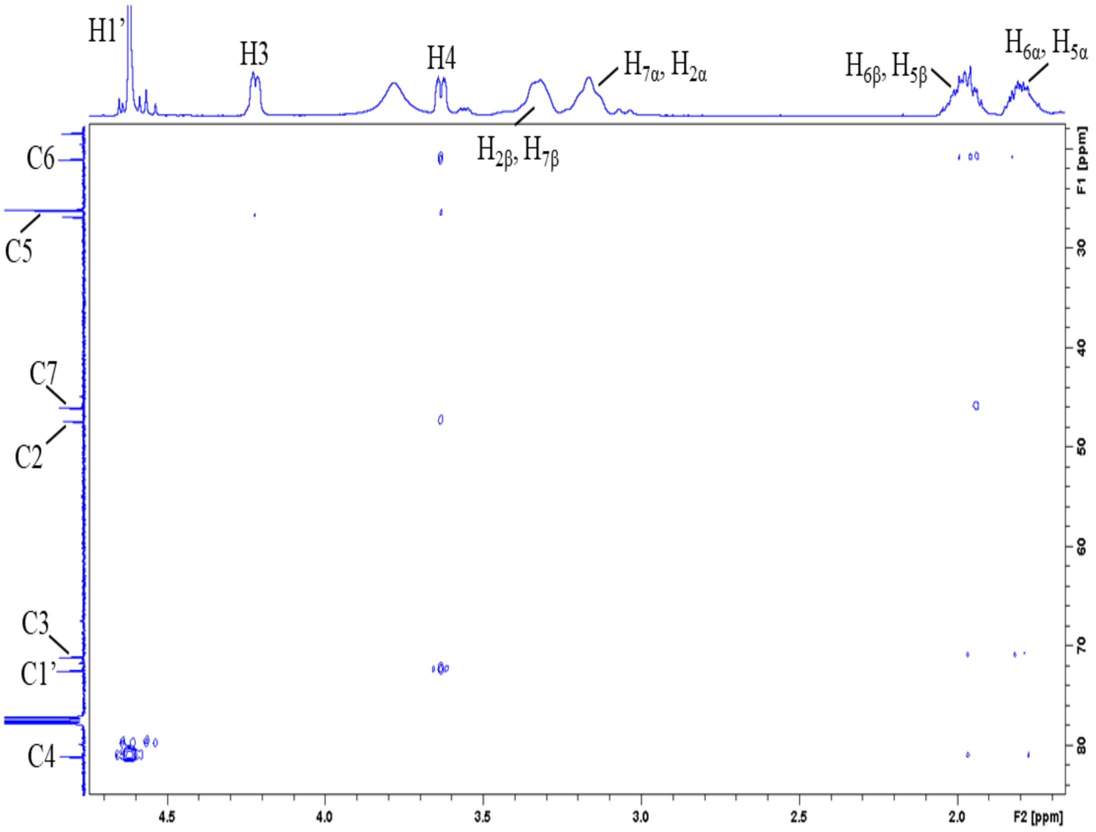

^{1}H-^{1}H COSY **4c**

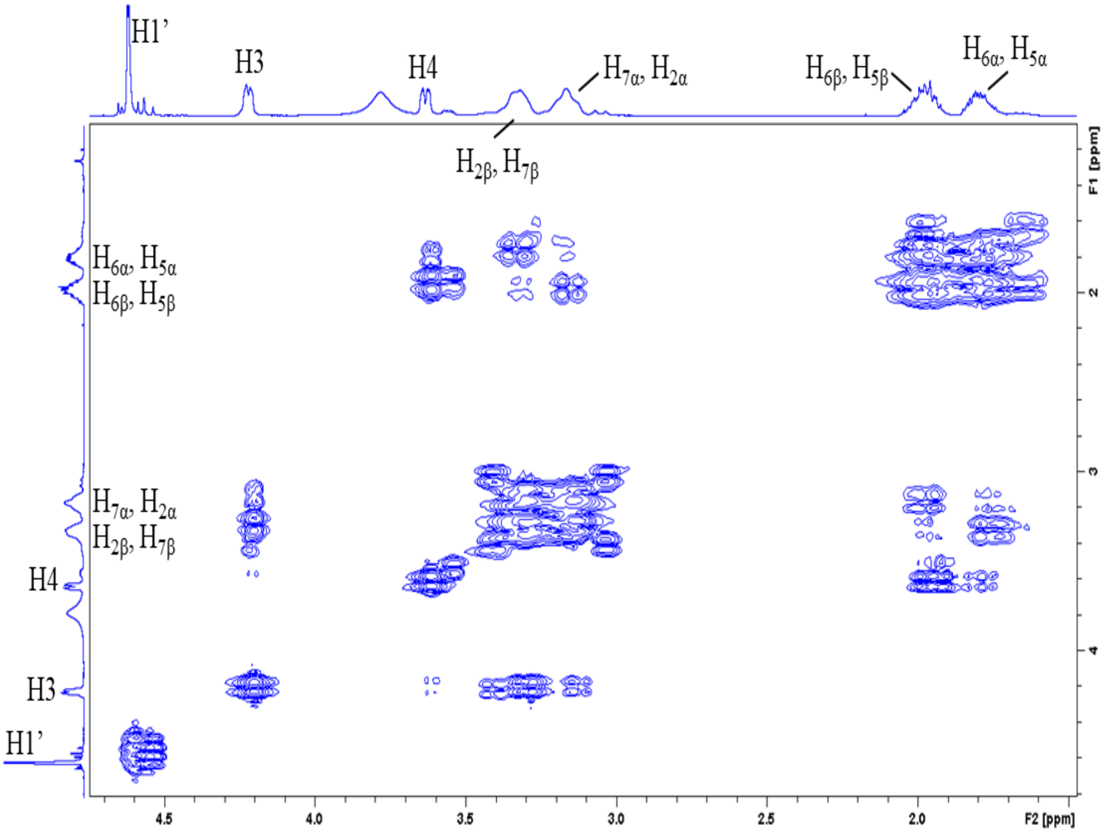

^{1}H-^{1}H NOESY **4c**

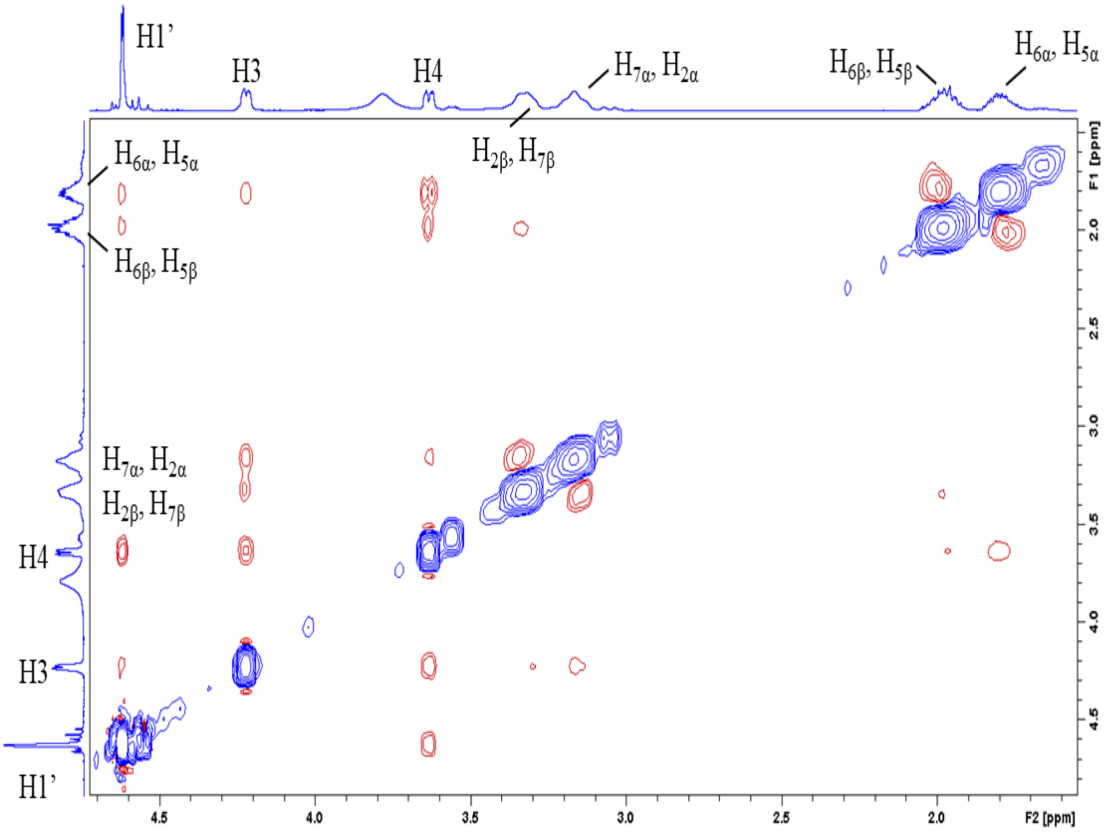

Structural Assignment of 6a

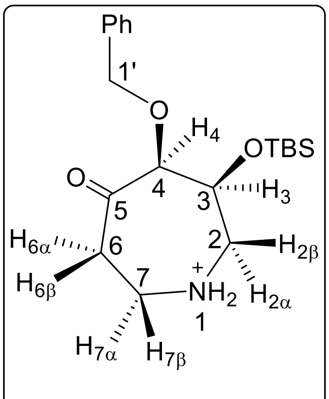

6a

Table S6. Structural assignment summary for 5-Oxoazepane **6a**

Pos.	δ_H, mult. (J in Hz)	δ_C	HMBC	COSY	NOESY
1′	4.48, d (11.6); 4.83, d (11.7)	73.4	4	-	3, 4
2α	3.30, dd (13.8, 2.2)	51.3	7	2β, 7α	2β, 3
2β	3.45 dd, (13.6, 6.2)		3, 4, 7	2α, 3, 4	2α, 3
3	4.28, dd (6.3, 1.9)	69.9	4, 5	2β, 4	2α/β, 4
4	4.32, s br	87.2	1′, 2, 3	2β, 3	1′, 2β, 6α/β, 7α
5	-	202.1	-	-	-
6α	2.78, t (5.9)	38.3	4, 5, 7	6β, 7α/β	4, 6β, 7α/β
6β	2.78, t (5.9)		4, 5, 7	6α, 7α/β	4, 6α, 7α/β
7α	3.26, dt (14.4, 6.3)	42.0	2, 5, 6	2α, 6α/β, 7β	4, 6α/β, 7β
7β	3.65, dt (14.5, 5.6)		2, 5, 6	6α/β, 7α	6α/β, 7α,

^{1}H-^{13}C HSQC **6a**

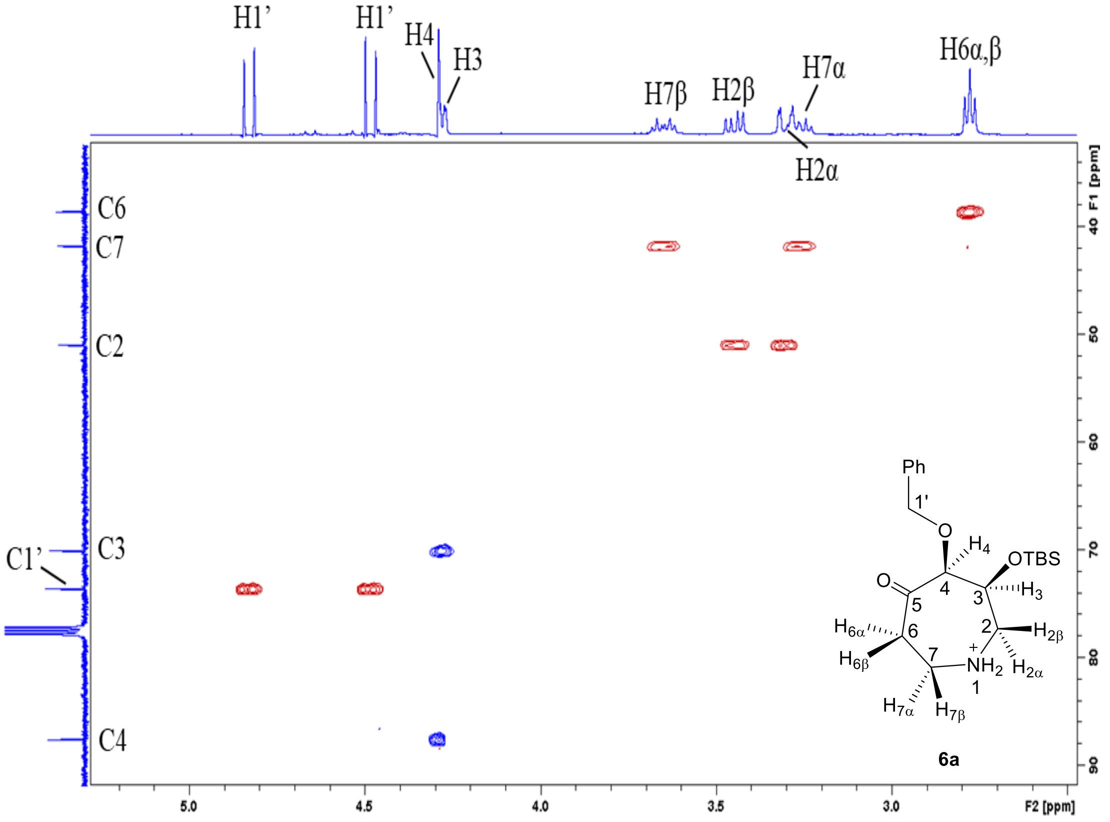

^{1}H-^{13}C HMBC **6a**

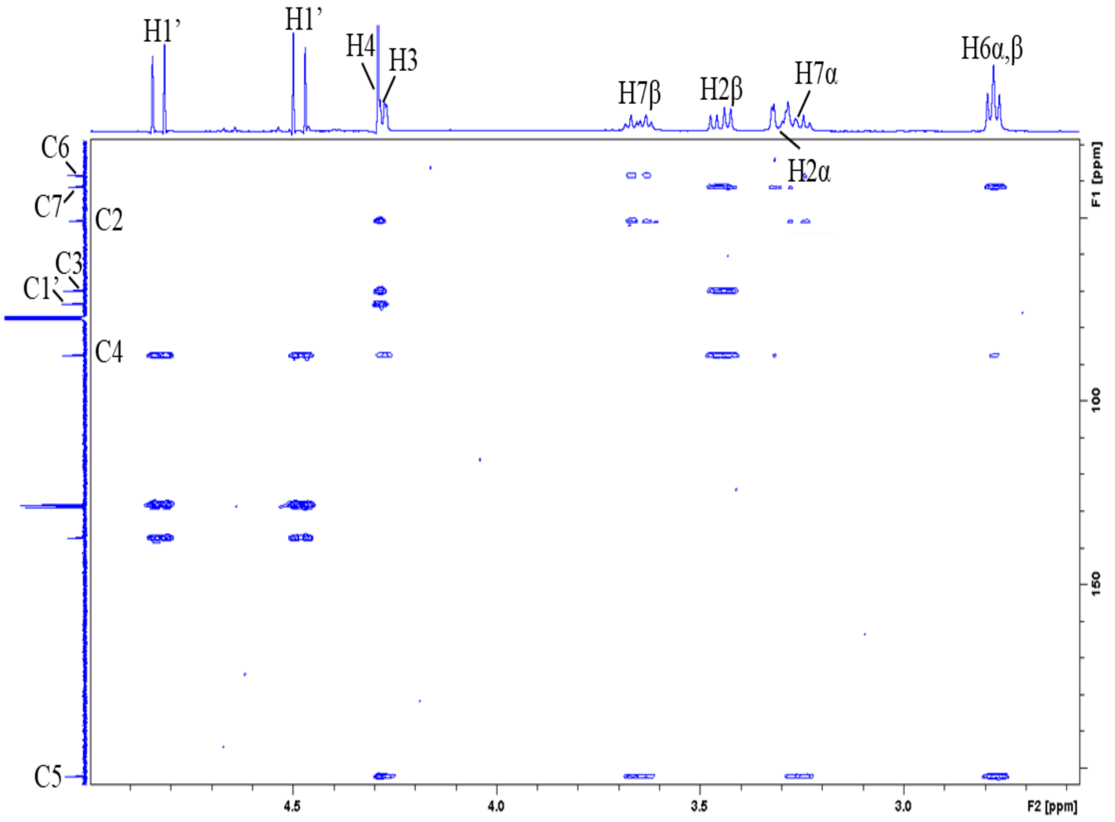

^{1}H-^{1}H COSY **6a**

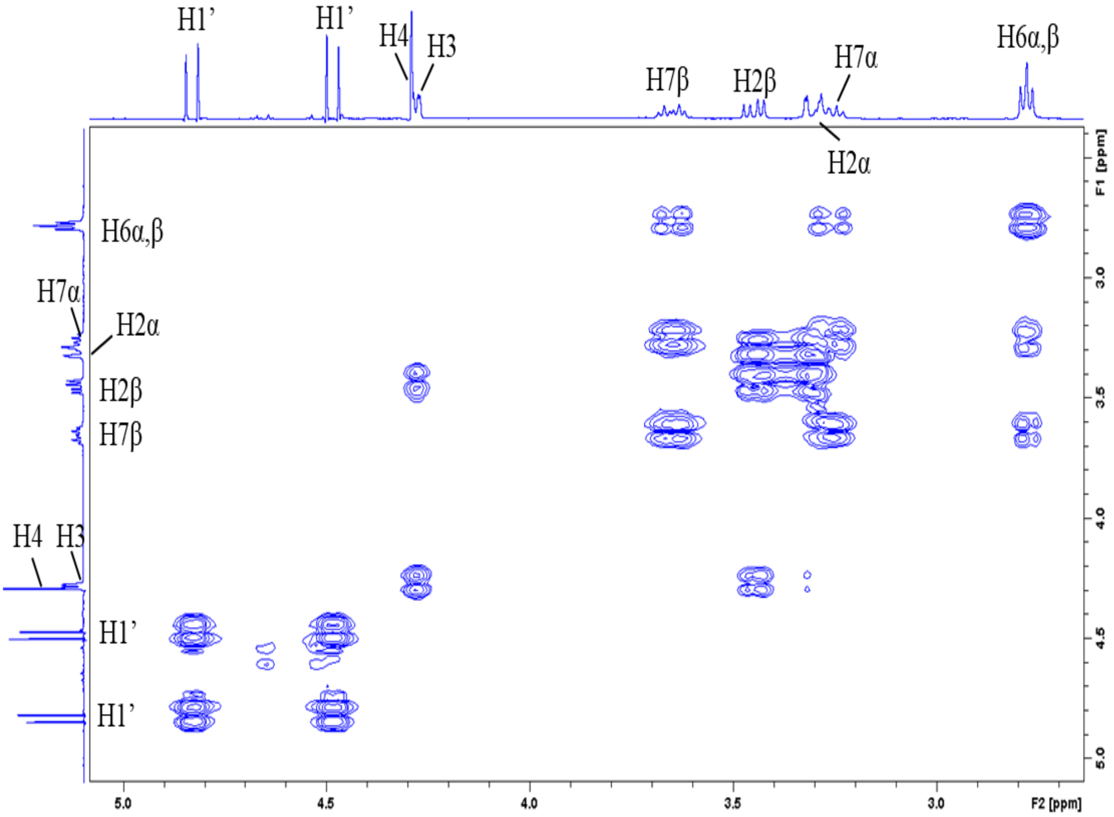

^{1}H-^{1}H NOESY **6a**

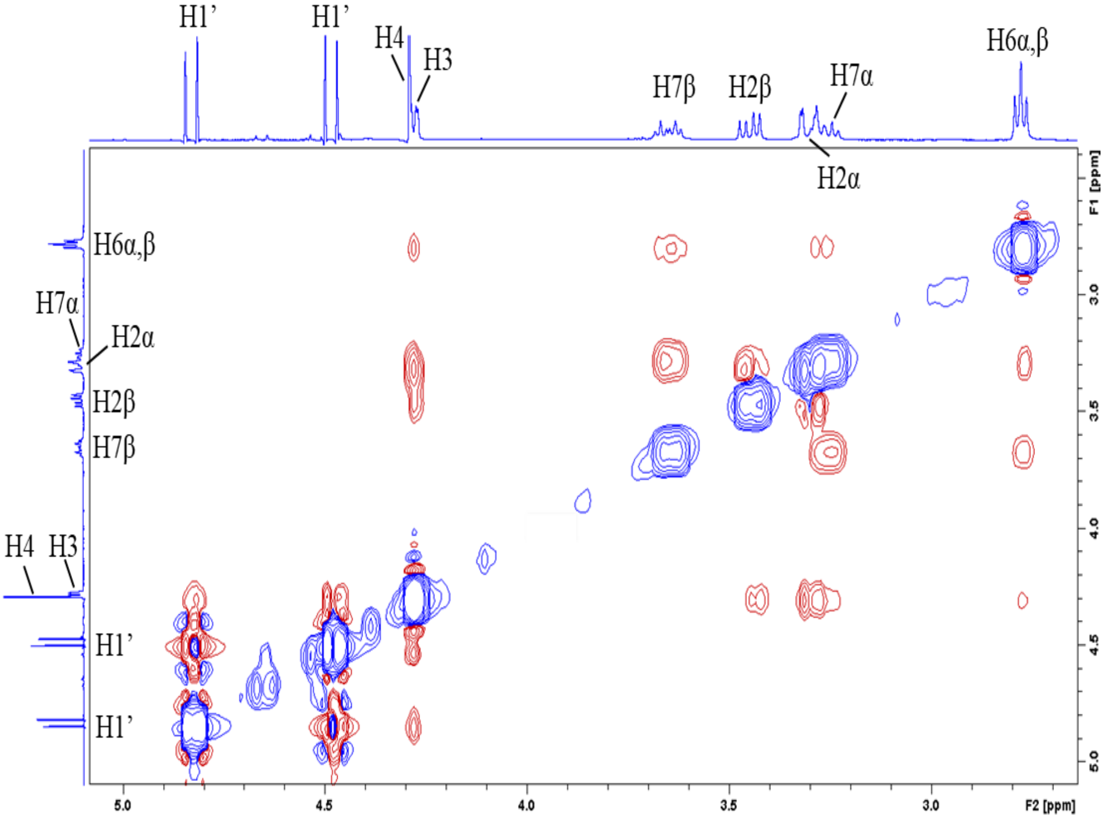

Structural Assignment of 6b

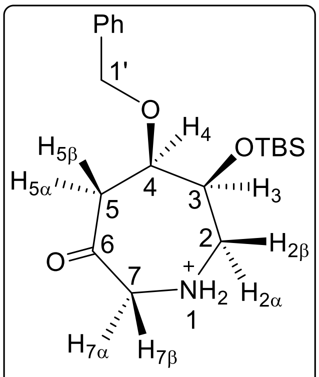

6b

Table S7. Structural assignment summary for 6-Oxoazepane **6b**

Pos.	δH, mult. (J in Hz)	δC	HMBC	COSY	NOESY
1′	4.67, s	73.2	4	-	4, 5α, 5β
2α	3.31, dd (13.2, 4.2)	49.1	3, 4, 7	2β, 3, 7β	2β, 3
2β	3.41, dd (13.1, 9.7)		3, 4, 7	2α, 3	2α, 3, 7β
3	4.32, ddd (9.6, 4.1, 1.8)	71.7	-	2α, 2β, 4	1′, 2α, 3
4	3.86, dt (7.4, 1.9)	75.5	1′, 2, 3, 6	3, 5α, 5β	1′, 2α, 3, 5α, 5β
5α	3.06, dd (15.3, 2.1)	43.5	3, 4, 6	4, 5β, 7α	3, 4
5β	3.12, dd (15.2, 7.3)		3, 4, 6, 7	4, 5α, 7α	1′, 4
6	-	200.5	-	-	-
7α	3.82, d (18.0)	56.4	2, 6	5β, 7β	2α, 7β
7β	3.65, d (18.0)		2, 6	2α, 7α	2β, 7α

^{1}H-^{13}C HSQC **6b**

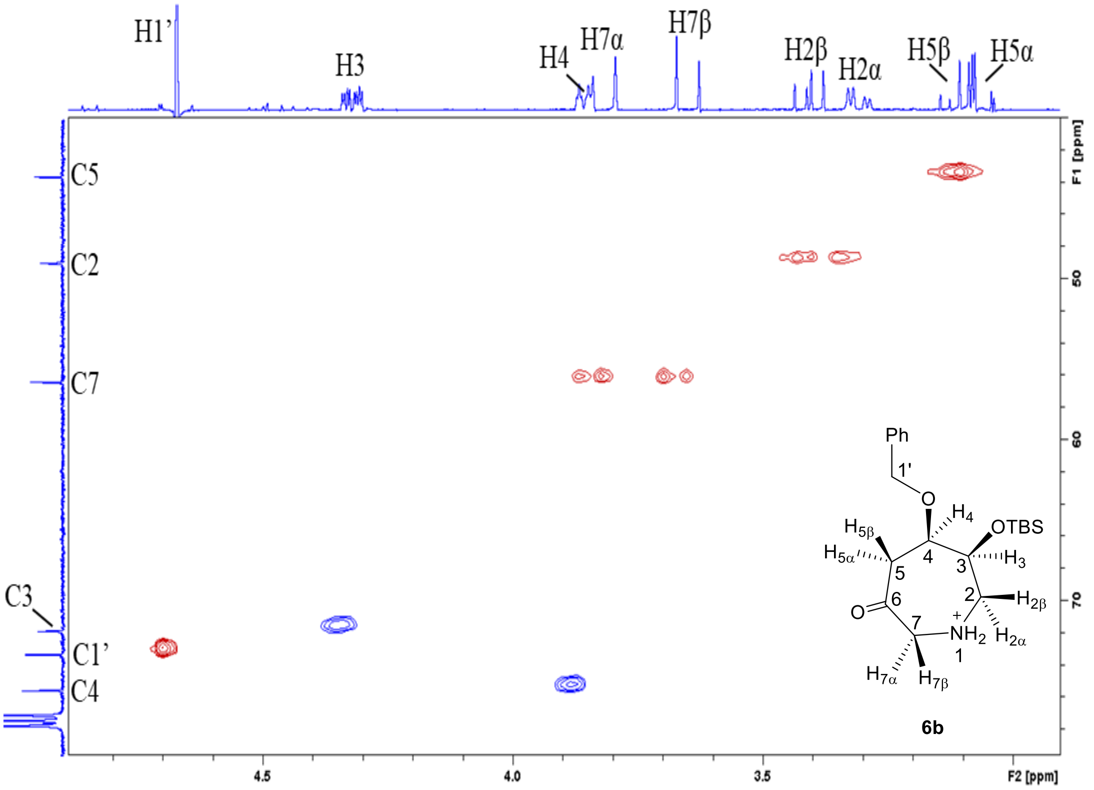

^{1}H-^{13}C HMBC **6b**

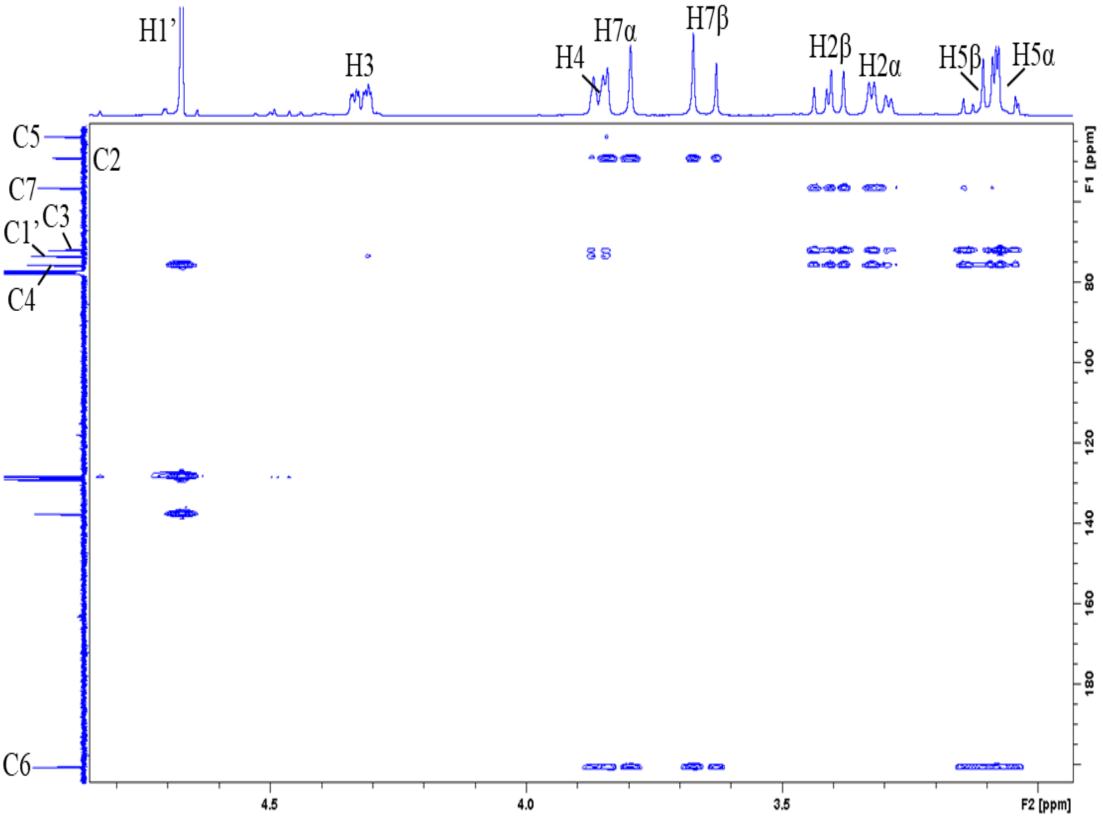

^{1}H-^{1}H COSY **6b**

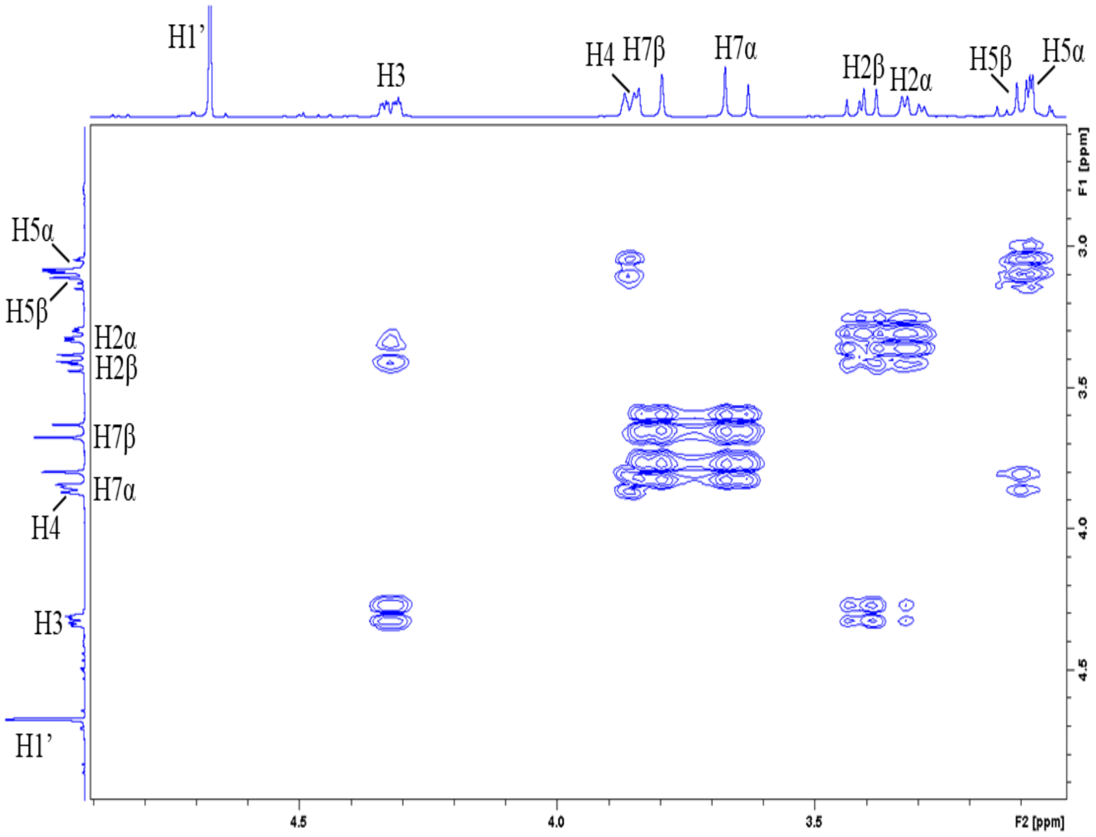

^{1}H-^{1}H NOESY **6b**

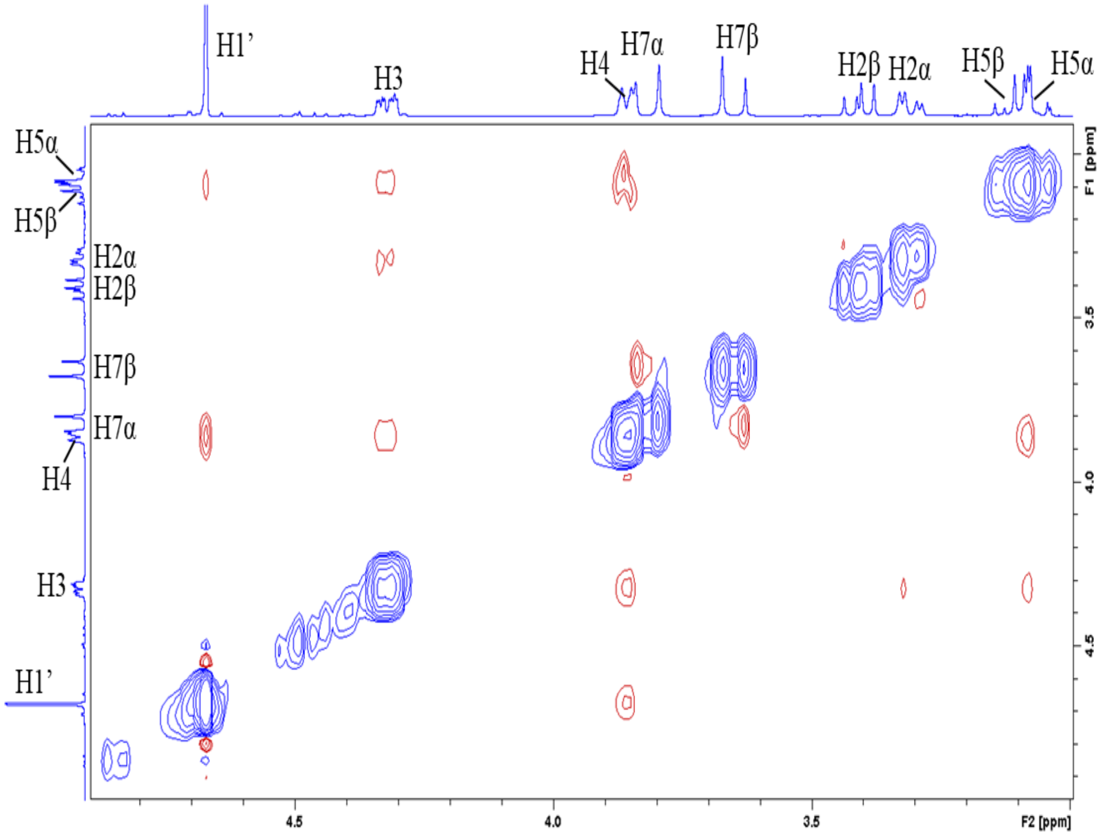

LC-MS Traces of Key Hydroboration Catalyst Screen Reactions

All analyses were conducted on a Phenomenex Gemini 3 μm, 110 Å 150x2 mm C18 column using UV detection (210 nm and 254 nm channels) and ESI-MS. A gradient of 50 to 80% acetonitrile in water over 40 minutes was used as the mobile phase. Sample concentration = 1 mg/mL.

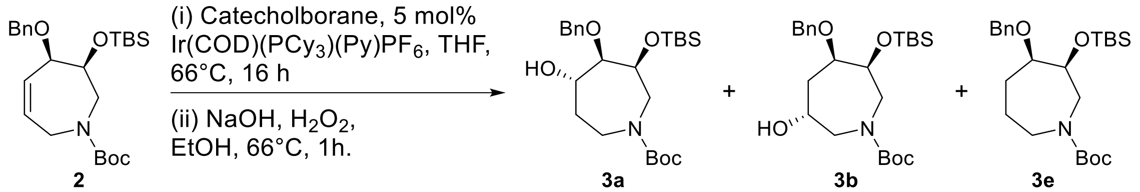

Table 2 - Entry 1

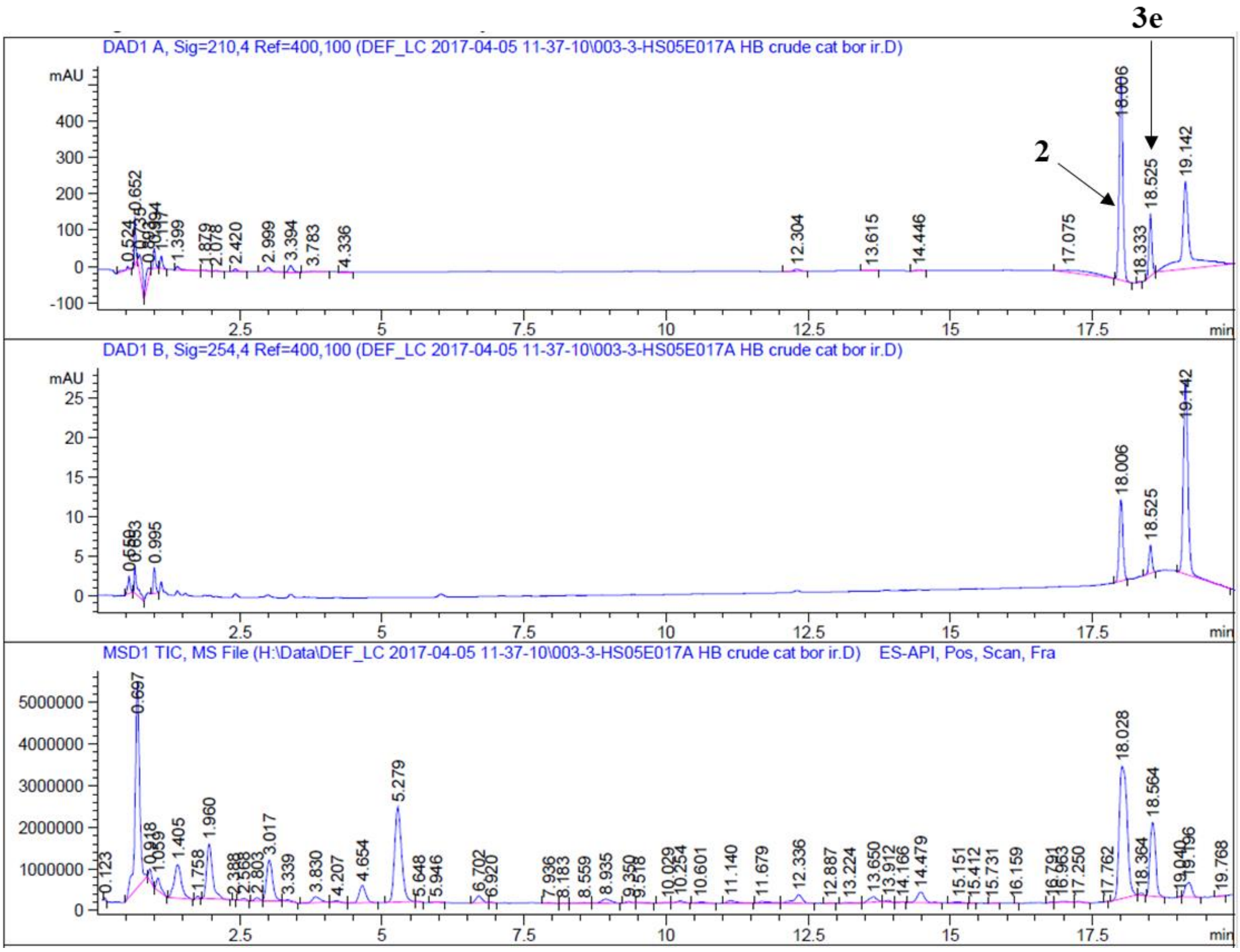

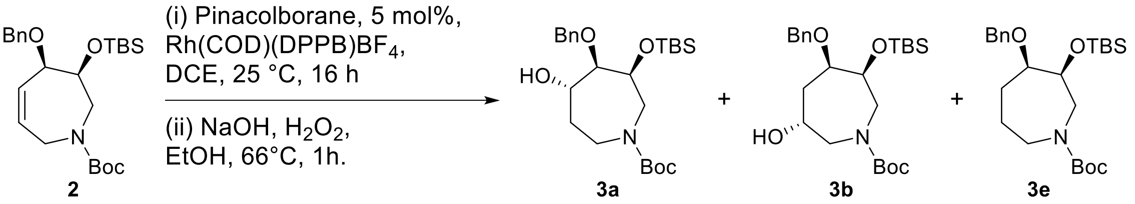

Table 2 – Entry 2

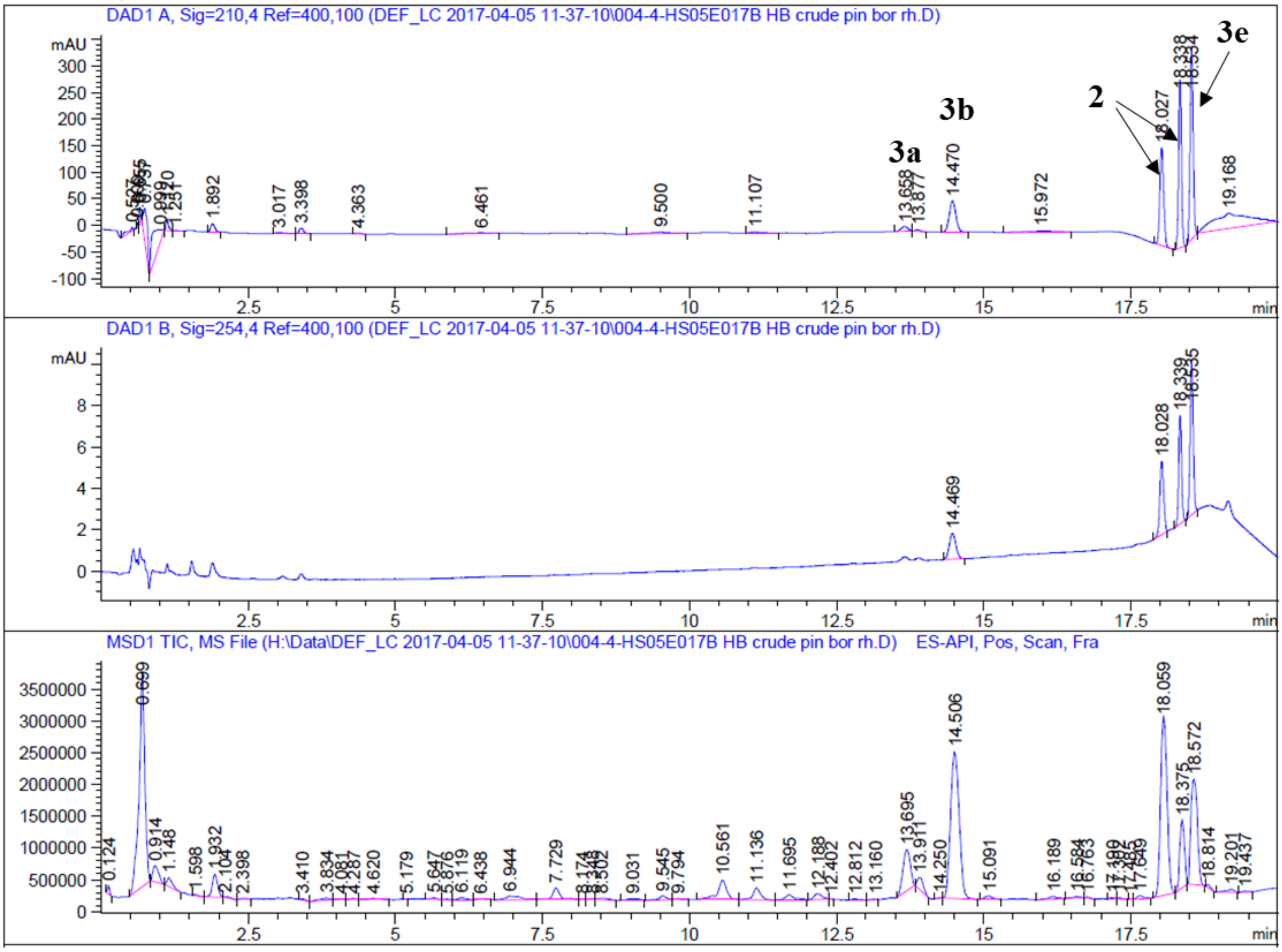

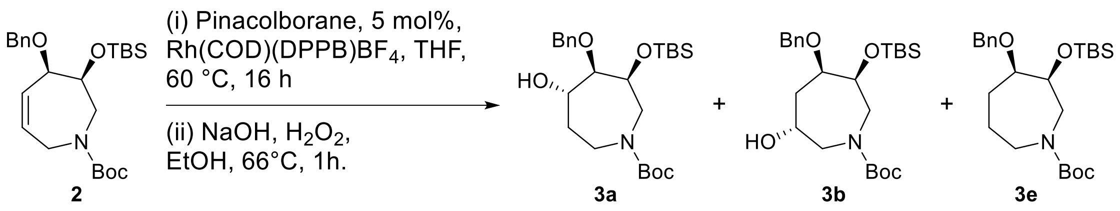

Table 3 – Entry 3

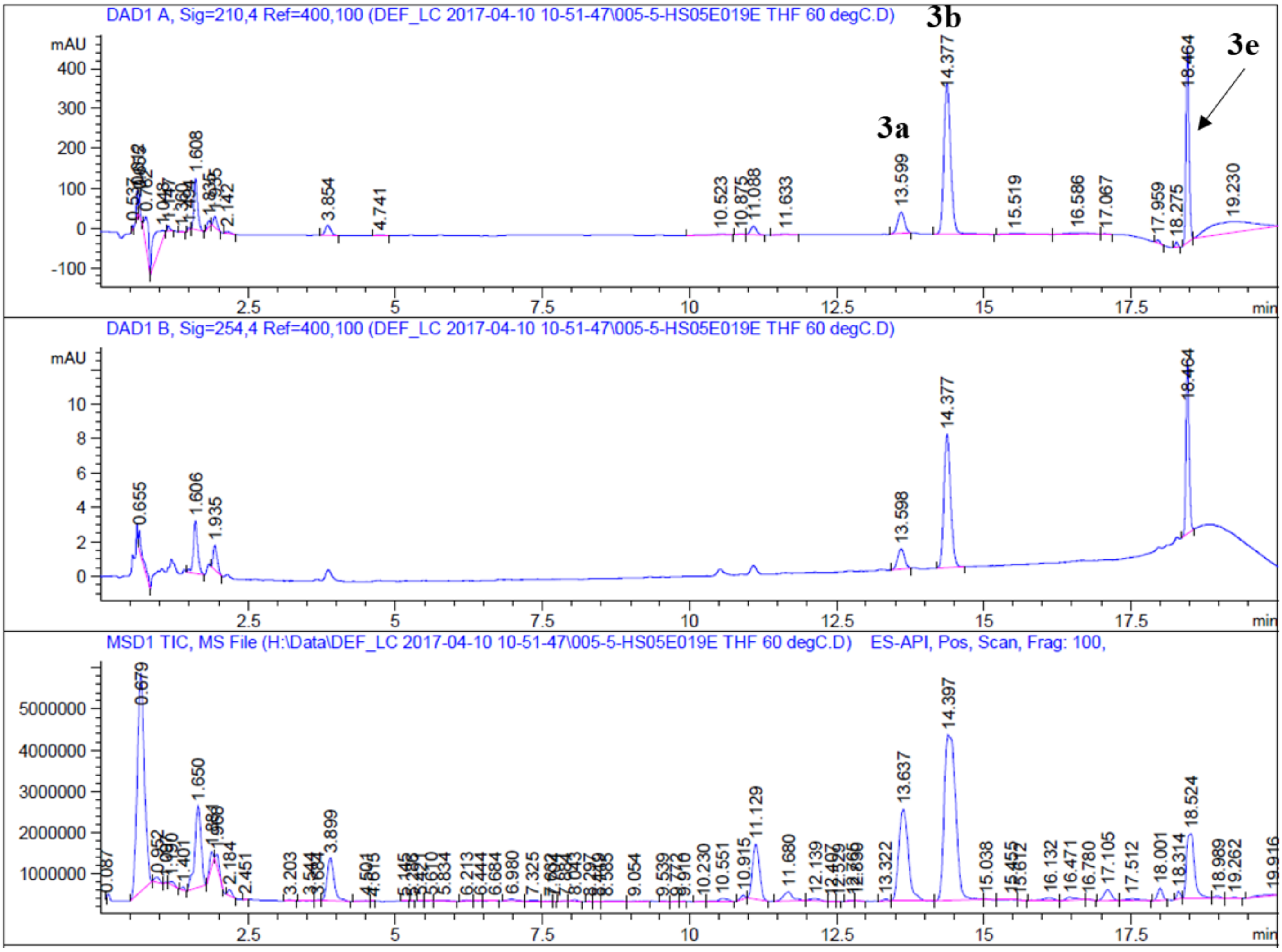

Comparative ¹H NMR Spectra of Hydrogenation Product 3e Versus Crude Reaction Mixtures

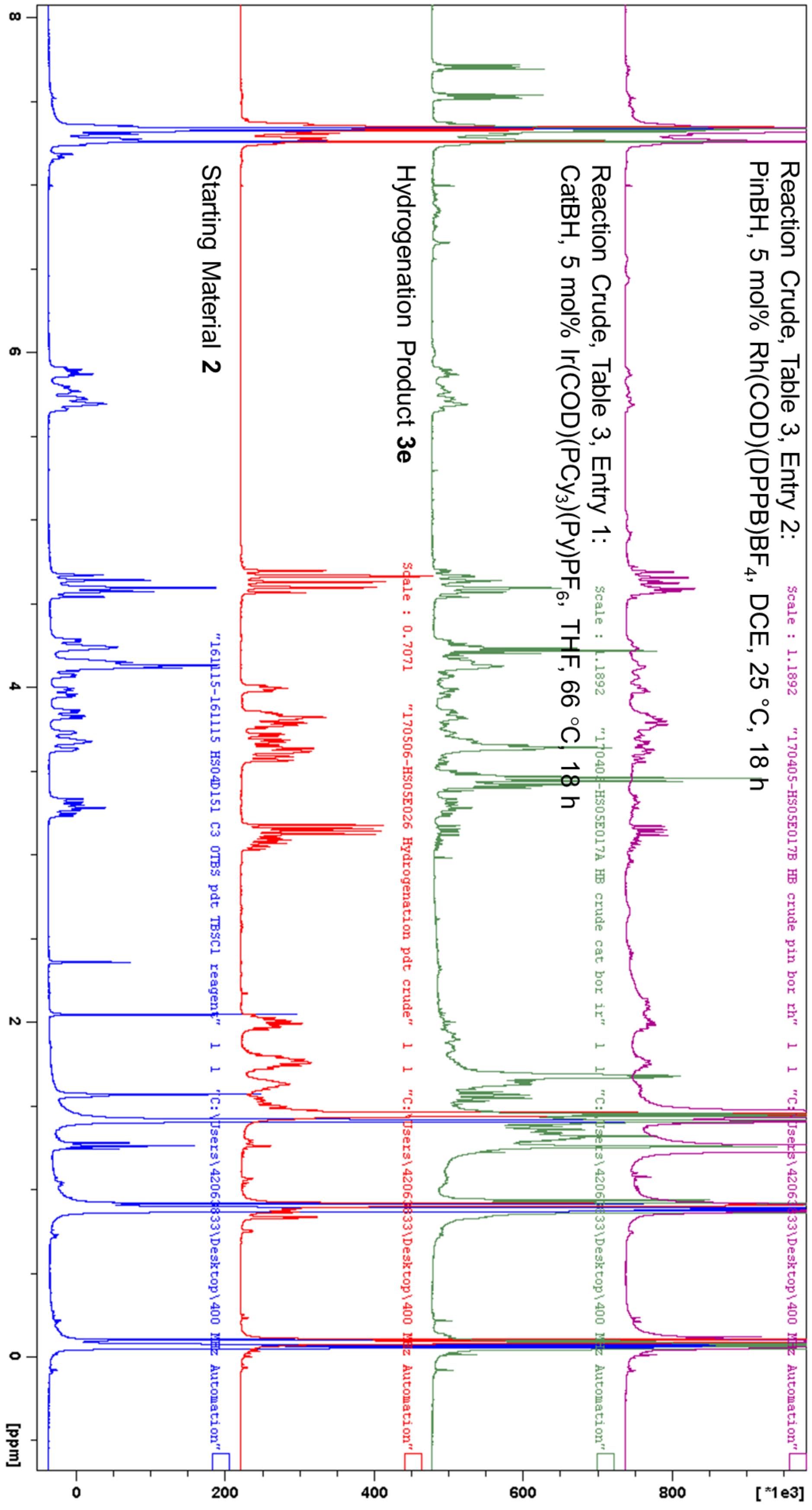

Supplement: Supplementary file 1 [file molecules-22-01871-s001.pdf]
